# Dissecting the Impact of Maternal Androgen Exposure on Developmental Programming through Targeting the Androgen Receptor

**DOI:** 10.1002/advs.202309429

**Published:** 2024-07-29

**Authors:** Haojiang Lu, Hong Jiang, Congru Li, Emilie Derisoud, Allan Zhao, Gustaw Eriksson, Eva Lindgren, Han‐Pin Pui, Sanjiv Risal, Yu Pei, Theresa Maxian, Claes Ohlsson, Anna Benrick, Sandra Haider, Elisabet Stener‐Victorin, Qiaolin Deng

**Affiliations:** ^1^ Department of Physiology and Pharmacology Karolinska Institutet Stockholm 17177 Sweden; ^2^ Department of Obstetrics and Gynaecology Reproductive Biology Unit, Placental Development Group Medical University of Vienna Vienna 1090 Austria; ^3^ Centre for Bone and Arthritis Research Department of Internal Medicine and Clinical Nutrition Institute of Medicine Sahlgrenska Academy University of Gothenburg Gothenburg 40530 Sweden; ^4^ Department of Physiology Sahlgrenska Academy University of Gothenburg Gothenburg 40530 Sweden; ^5^ School of Health Sciences University of Skövde Skövde 54128 Sweden

**Keywords:** developmental programming, human trophoblast organoids, placental development, polycystic ovary syndrome

## Abstract

Women with polycystic ovary syndrome (PCOS) exhibit sustained elevation in circulating androgens during pregnancy, an independent risk factor linked to pregnancy complications and adverse outcomes in offspring. Yet, further studies are required to understand the effects of elevated androgens on cell type‐specific placental dysfunction and fetal development. Therefore, a PCOS‐like mouse model induced by continuous androgen exposure is examined. The PCOS‐mice exhibited impaired placental and embryonic development, resulting in mid‐gestation lethality. Co‐treatment with the androgen receptor blocker, flutamide, prevents these phenotypes including germ cell specification. Comprehensive profiling of the placenta by whole‐genome bisulfite and RNA sequencing shows a reduced proportion of trophoblast precursors, possibly due to the downregulation of *Cdx2* expression. Reduced expression of *Gcm1*, *Synb*, and *Prl3b1* is associated with reduced syncytiotrophoblasts and sinusoidal trophoblast giant cells, impairs placental labyrinth formation. Importantly, human trophoblast organoids exposed to androgens exhibit analogous changes, showing impaired trophoblast differentiation as a key feature in PCOS‐related pregnancy complications. These findings provide new insights into the potential cellular targets for future treatments.

## Graphical abstract

Women with polycystic ovary syndrome (PCOS) encounter a higher miscarriage rate. A mouse model mimicking maternal PCOS is generated searching for the underlying mechanism. Excessive androgen exposure during pregnancy is found to cause embryonic lethality during mid‐pregnancy due to malformation of the placenta, especially the nutrient exchange labyrinth zone. Disrupted of trophoblast cell types is also found in human trophoblast organoids treated with androgen.

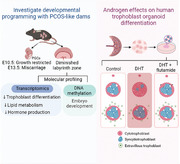

## 1 Introduction

Polycystic ovary syndrome (PCOS) occurs in 10–13% of women during reproductive age, and leads to subfertility characterized by irregular menstrual cycles, diminished endometrial receptivity, and endometrial cancer.^[^ ^1^ ^]^ Additionally, PCOS is associated with comorbidities such as type 2 diabetes, and psychiatric morbidity.^[^ ^2^ ^]^ Moreover, individuals with PCOS encounter elevated risk of early pregnancy loss, preeclampsia, preterm delivery,^[^ ^3^ ^]^ and adverse neonatal outcomes.^[^ ^4^ ^]^ These obstetric challenges confer a predisposition upon offspring to develop reproductive and cardiometabolic disorders in adulthood.^[^ ^1^, ^5^, ^6^ ^]^

The placenta, a transient organ, facilitates the supply of oxygen and nutrients to the developing fetus throughout the entire pregnancy. Despite the differences in physiological and morphological features, both human and mouse placentas exhibit a hemochorial structure, wherein the fetal trophoblast establishes direct contact with maternal blood circulation.^[^ ^7^ ^]^ The trophoblast lineage consists mainly of cytotrophoblast cells, which give rise to syncytiotrophoblasts in both species, and invasive extravillous trophoblasts in humans as the counterpart to the giant cells of the mouse trophoblast. Syncytiotrophoblasts play a pivotal role in placental steroidogenesis and hormone production, while invasive trophoblast cells are crucial for the successful establishment of pregnancy.^[^ ^8^ ^]^ Previous studies have demonstrated that dysfunction in any of these cell types can lead to adverse outcomes such as pre‐eclampsia, uterine growth restriction, or even miscarriage.^[^ ^7^, ^9^ ^]^ Similarly, restoration of essential placental gene expression, resulting in the replenishment of critical placental cell types, has been shown to prevent embryo lethality.^[^ ^10^, ^11^ ^]^

Women with PCOS have a persistent increase in circulating androgens and anti‐Müllerian hormone (AMH) levels throughout pregnancy,^[^ ^12^, ^13^ ^]^ which has been shown to be an independent risk factors for pregnancy complications and adverse neonatal outcomes. Notably, women with PCOS manifest an unfavorable maternal‐fetal environment characterized by aberrant placenta morphology, diminished invasion sites, and impaired steroidogenesis activities,^[^ ^14^, ^15^, ^16^ ^]^ thereby impacting fetal development. Nevertheless, the specific mediation of these adverse effects and the impact on different cell types of placental trophoblasts in the context of maternal hyperandrogenism need to be further investigated.

We and others have demonstrated that maternal hyperandrogenism exerts harmful effects on fetal development,^[^ ^17^, ^18^ ^]^ consequently predisposing their offspring to subsequent reproductive, metabolic, and psychiatric disorders.^[^ ^19^, ^20^, ^21^ ^]^ Specifically, daughters born to women with PCOS exhibit a fivefold increase in the likelihood of being diagnosed with PCOS,^[^ ^6^ ^]^ and are prone to develop psychiatric disorders later in life.^[^ ^22^ ^]^ Additionally, newborns display an elongated anogenital distance (AGD), a strong marker of in utero androgen excess.^[^ ^6^, ^23^ ^]^ Conversely, sons born to women with PCOS face an elevated risk of developing obesity, hyperlipidemia, anxiety, and depression.^[^ ^5^, ^24^, ^25^ ^]^ Previous studies have shown that activation of the androgen receptor in androgenized pregnant rats results in altered implantation and uterine mitochondrial dysfunction, as well as diminished decidualization and angiogenesis which may mediate the effects on offspring.^[^ ^17^ ^]^ However, whether and how maternal androgen exposure affects the placenta, early embryo, and primordial germ cell development, and whether co‐treatment targeting the androgen pathways has the potential to prevent placenta dysfunction leading to normal fetal and germ cell development remains to be investigated.

We here present evidence that an in utero hyperandrogenic environment in mice profoundly compromises both placental, embryo and germ cell development, which can be prevented by administration of the androgen receptor blocker, flutamide. Using whole genome bisulfite sequencing (WGBS) and RNA sequencing, we identified molecular alterations in placentas and primordial germ cells (PGCs) at critical embryonic developmental time points, specifically embryonic days (E)10.5 and E13.5. We revealed significant impairment in the differentiation of trophoblast cell lineages within the placenta, culminating in miscarriage in female mice exposed to androgens during pregnancy. Additionally, disruptions in placental fatty acid metabolism were identified suggesting a potential predisposition of the offspring to metabolic abnormalities later in life. The impairment in trophoblast differentiation and invasion capacity was also demonstrated in the human trophoblast organoids exposed to androgens. And flutamide also ameliorated such impairments. These results provide novel insights into the complications associated with PCOS during pregnancy and thus provide information for the development of future treatments.

## 2 Results

### 2.1 Flutamide Mitigates Reproductive Dysfunction Induced by Androgen Exposure

**Figure 1:**
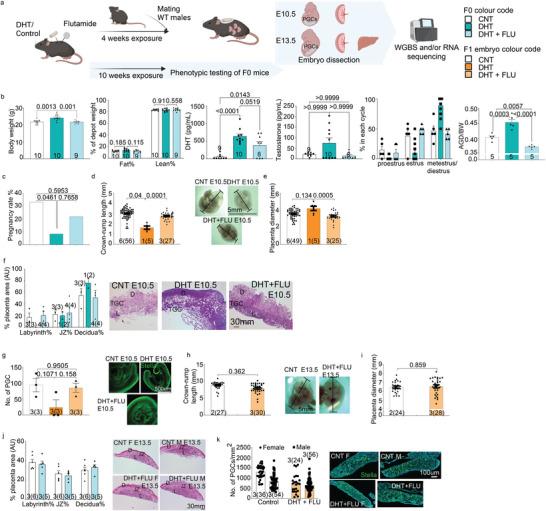
Peripubertal androgen‐induced PCOS‐like mouse model negatively affects pregnancy outcomes that are prevented by co‐treatment with flutamide. a) Schematic overview of the experimental design and breeding scheme of the embryonic part. Control = vehicle; DHT = implanted with slow‐releasing 10 mm DHT pellet; DHT + FLU = implanted with 10 mm DHT pellet and a flutamide pellet. b) Peripubertal PCOS‐like (F0) mice weigh more (Control = 10, DHT = 10, DHT + FLU = 9) but showed similar relative fat and lean mass, have increased circulating DHT levels measured by LC‐MS, disrupted estrus cyclicity (*P* < 0.05 for M/D phase CNT versus DHT and DHT versus FLU comparison; CN T = 5, DHT = 15, DHT + FLU = 5), longer anogenital distance (AGD), and developed typical polycystic ovarian morphology according to Hemotoxilin and Eosin (H&E) stains. c) Pregnancy rate and number of embryos per F0 dam; (Control = 40, DHT = 40, DHT + FLU = 28). d) Crown‐rump length and representative images of E10.5 embryos from each group. e, Placenta diameter measured at the time of dissection. f) Measurement of different placental zone areas normalized by total placenta area and representative bright‐field images of H&E staining of E10.5 placentas. g) Number of PGCs counted in fixed E10.5 embryos, 1 embryo per dam is taken for this purpose; Control = 3, DHT = 3, DHT + FLU = 3. And representative confocal microscope images of E10.5 whole mount embryo staining of PGCs with Stella. h) Crown‐rump length, and representative images of E13.5 embryos from each group. i) Placenta diameter measured at the time of dissection. j) Measurement of different placental zone areas normalized by total placenta area and representative bright‐field images of H&E staining of E13.5 embryo placentas. k) Number of primordial germ cells (PGCs) counted in fixed E13.5 embryo gonads; Control = 3/gender, DHT + FLU = 3/gender, and representative confocal microscope images of E13.5 gonads with PGCs stained with Stella. TGC: trophoblast giant cell, L: labyrinth zone, JZ: junctional zone, D: decidua. All data are presented as mean ± SEM. Numbers of dams and P value are stated in the text of graph or in the bar of each group whenever possible, the number of embryos was given in the bracket. F0: One‐way ANOVA with Dunnet post hoc analysis if passed the Shapiro Wilk normality test; otherwise Kruskal‐Wallis test is used. F1 embryos: use of ANCOVA to account for litter size whenever possible otherwise One‐way ANOVA with Dunnet post hoc analysis was used.

A PCOS‐like mouse model was established by implantation of dihydrotestosterone (DHT) pellets into 4‐week‐old peripubertal F0 female mice,^[^ ^26^, ^27^ ^]^ hereafter referred to as PCOS‐mice, in comparison to control mice implanted with inert pellets. To investigate the potential causal involvement of the androgen receptor pathway in the development of PCOS‐like traits and to assess the preventive capacity of downstream effects, one group of mice received simultaneous implantation of a DHT‐pellet and a continuous slow‐releasing flutamide pellet, an androgen receptor antagonist, hereafter referred to as flutamide mice (**Figure** **1**a). Phenotypic assessments were conducted in all F0 mice after 10 weeks of exposure.

Following 10‐weeks of DHT exposure, the PCOS‐mice exhibited an increase in body weight compared to controls, while the flutamide‐mice were protected against weight gain (one‐way ANOVA: *F*_2, 26_ = 10.42; *P* = 0.0005) (Figure 1b). Despite the significant weight gain in the PCOS‐mice, they have comparable relative fat and lean mass with control or flutamide treated mice as analyzed by body composition measured by EchoMRI (Figure 1b). Circulating DHT level was higher in both PCOS‐mice and flutamide‐mice (one‐way ANOVA, *F*_2, 24_ = 16.82; *P* = 0.024) (Figure 1b), with no discernable difference in circulating testosterone level between the groups (Kruskal Willis test, *H* = 2.833; *P =* 0.243) (Figure 1b). Although not significant, the two‐to‐three‐fold increase in circulating testosterone level induced by DHT pellet implantation resembles the fold change in women with PCOS.^[^ ^28^ ^]^ Reproductive function was evaluated through a 12‐day continuous vaginal smear, revealing that the PCOS‐mice were consistently in metestrus/diestrus phase indicating complete anovulation. In contrast, control‐ and flutamide‐mice exhibited normal cyclicity (Figure 1b). Elongated anogenital distance (AGD), a robust indicator of androgen exposure, was evident in PCOS‐mice compared to controls, and this effect was prevented by flutamide treatment (one‐way ANOVA: *F*_2,12_ = 38.81; *P* = <0.0001) (Figure 1b). These findings affirm the successful establishment of PCOS‐like mouse model through peripubertal DHT pellet implantation and underscore the androgen receptor pathways as mediators of the observed phenotypic traits.

### 2.2 Flutamide Mitigates Abnormal Placental and Embryonic Development Induced by Maternal Androgen Excess

To comprehensively assess the impact of maternal androgen excess on placental function and embryonic development, we used the peripubertal DHT‐induced PCOS mouse model with or without flutamide pellets to interrogate the effect of androgen receptors activation. Following 4 weeks of exposure, these mice were mated with wildtype males, necessitating superovulation due to anovulatory feature of the PCOS‐mice. A total of 40 PCOS‐mice, 40 control female mice (F0), and 28 flutamide‐treated PCOS‐mice were used for this purpose. Embryos were collected and dissected at E10.5 and E13.5, two critical stages to examine the early‐ and mid‐embryonic development and the dynamics of embryo DNA methylation (Figure 1a).

The PCOS‐mice (F0) exhibited a diminished pregnancy rate compared to the controls, an effect that was completely prevented by flutamide (Kruskal‐Wallis: *H* = 5.882; *P* = 0.041) (Figure 1c). Additionally, the quantification of deceased/absorbed embryos per dam at the time of dissection revealed a higher count in the PCOS‐mice, a phenomenon that was also normalized by flutamide co‐treatment at both E10.5 and E13.5 (E10.5: Kruskal‐wallis test, *H* = 7.017; *P =* 0.016; E13.5: student t test, *P >* 0.999) (Figure S1a, Supporting Information). To investigate potential sex‐specific embryo loss, live embryos of each sex were recorded in Table S1 (Supporting Information). No significant difference was observed in the sex ratio among the surviving embryos at E10.5 (effect of sex: F(1,14) = 2.137, P = 0.166; effect of group: F(2,14) = 0.000, P > 0.999) and E13.5 (effect of sex: F(1,8) = 1.026, P = 0.341; effect of group: F(1,8) = 0.000, *P* > 0.999).

In addition to the diminished pregnancy rate observed in the PCOS‐mice, embryos were smaller, as indicated by a shorter crown‐rump length at E10.5. Notably, this effect was absent in the flutamide‐treated group (ANCOVA: *P* = 0.04) (Figure 1d). PCOS‐mice exhibited a trend toward larger placenta compared to control and flutamide groups (ANCOVA: *P_CNT_*_versus_ *_DHT_* = 0.134, *P_DHT_*_versus_ *_DHT + FLU_* = 0.0005) (Figure 1e). Morphological examination of the PCOS‐mice placenta at E10.5 revealed aberrant organization of the labyrinth layer characterized by a diminished labyrinth area and increased but disorganized trophoblast giant cells, while the relative area of decidua and junctional zone appeared to be less affected. In contrast, the flutamide‐mice showed comparable placental morphology and relative size of the different layers as the control mice (Figure 1f; Figure S1b, Supporting Information). Additionally, the embryos from PCOS‐mice exhibited a smaller number of migrating PGCs at E10.5, albeit insignificant, it was prevented by flutamide treatment (ANCOVA: *P* = 0.107) (Figure 1g). Whole‐mount staining of the embryo at E10.5 also revealed a delay in both embryo and PGC development, evident from the migratory PGC location (Figure 1g). All embryos from PCOS‐mice failed to develop beyond E13.5. Consequently, only embryos from control and flutamide‐treated dams were available for analyses at E13.5. Notably, E13.5 embryos from the flutamide‐mice were similar to those of controls, with no difference in crown‐rump length, placental size, the number of migrating PGCs, or the placenta morphology in both sexes (Figure 1h–k; Figure S1c, Supporting Information). Collectively, these findings underscore the pivotal role of androgen receptor pathway activation in mediating maternal effects in PCOS.

### 2.3 Androgen Exposure Alters Multiple Signaling Pathways Important for Placenta Function

**Figure 2:**
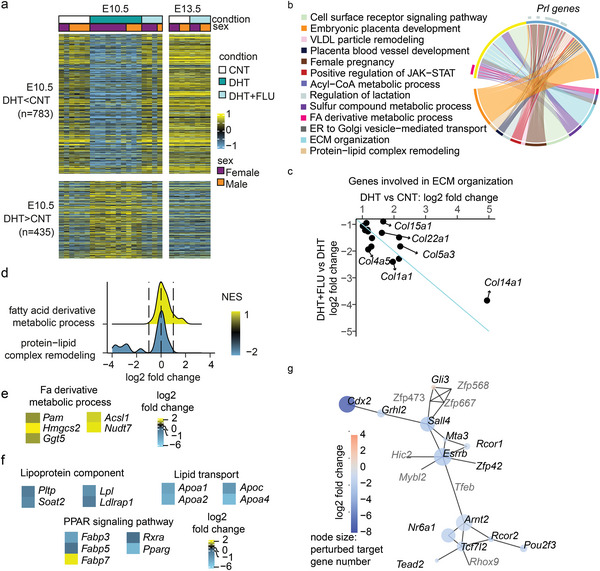
Altered placental signaling pathways by maternal hyperandrogenism are prevented by flutamide treatment. a) Heatmap displaying the expression pattern of 1218 DEGs (differentially expressed genes) in PCOS‐mice compared to control‐mice at E10.5. The color bar indicates the scaled log2 normalized counts. b) Selected cellular process gene ontology pathways enriched by DEGs in PCOS‐mice compared to control‐mice at E10.5. The pathways shown are enriched using clusterProfiler package (v4.0.5). In the upper half of the sphere, the yellow region marks up‐regulated genes, and the blue marks the down‐regulated genes. The lower half shows the enriched pathways. The arcs link the genes to their related pathways. c) Scatter plots of fold changes of the genes involved in the extracellular matrix organization estimated with DESeq2. d) The gene set enrichment with the distribution of log2 fold change and normalized enrichment scores. e) Transcription analysis of E10.5 CNT versus PCOS placenta DEGs involved in GO term “fatty acid derivative metabolic process”. f) Downregulated gene expression of DEGs involved in lipid transport, lipid storage (lipoprotein component), and PPAR (Peroxisome proliferator‐activated receptor) signaling pathway. The gene set of each metabolic process to intersect with the DEGs is from KEGG database and the enrichment is by a hypergeometric test with q‐value < 0.05. g) Co‐expression network of differentially expressed transcription factors in placentae from DHT‐mice. The edges indicate the co‐expression (median confidence > = 0.4). The node size indicates the number of its target genes which were DEGs in PCOS‐mice (min: 0, max: 965). The grey gene names were those without a record in TFlink database, thus, they got zero sizes. Number of dams used for RNA sequencing analysis: For embryonic stage E10.5, CNT = 3, DHT = 1, DHT+FLU = 3. For embryonic stage E13.5, CNT = 3, DHT+FLU = 3. Data are presented as log2 fold change, and all genes displayed were DEGs by DESeq2.

To investigate the molecular profile of placentas derived from PCOS‐mice compared with control or flutamide‐mice (Figure S2a and Tables S2 and S3, Supporting Information) bulk RNA sequencing technique was applied. At E10.5, a total of 783 genes showed decreased expression, while 435 genes exhibited increased expression (**Figure** **2**a; Figure S2a,b and Table S4, Supporting Information). Notably, placentas from flutamide‐mice displayed a transcriptional profile indistinguishable from that of control mice at both at E10.5 and E13.5 (Figure 2a; Figure S2a, Supporting Information), indicating that the inhibition of the androgen receptor safeguarded against the transcriptional perturbation observed in PCOS‐mice.

Enriched Gene Ontology Biological Process revealed that maternal androgen exposure reduced the expression of genes involved in multiple signaling pathways associated with female pregnancy, including placental development and the regulation of lactation (Figure 2b; Figure S2c, Supporting Information). Maternal hyperandrogenism also enhanced several signaling pathways, including extracellular matrix organization and PPAR‐regulated fatty acid metabolism. In the extracellular matrix, collagens are the predominant structural proteins. The expression of genes encoding members of the collagen family was increased in the PCOS placentas and flutamide cotreatment almost completely reversed the expression change (Figure 2c). Another elevated pathway in PCOS placentas is the fatty acid and the associated acyl‐CoA metabolic process which could be associated with the increased adiposity observed in PCOS (Figure 2b,d). The upregulation of *Acsl1*, encoding an enzyme responsible for synthesizing fatty acyl‐CoAs from long‐chain‐fatty‐acid,^[^ ^29^ ^]^ and *Nudt7*, encoding an enzyme involved in the hydrolysis of fatty acyl‐CoAs,^[^ ^30^ ^]^ was observed in PCOS‐mice (Figure 2e). Nonetheless, the protein−lipid complex remodeling pathway was downregulated in PCOS‐mice placentas (Figure 2d).^[^ ^29^ ^]^ Specifically, *Hmgcs2*, encoding HMG‐CoA synthase, involved in a metabolic pathway providing lipid‐derived energy,^[^ ^31^ ^]^ exhibited a four‐fold increase in expression in PCOS‐mice compared to controls (Figure 2e). In parallel, a gene set enrichment analysis of pathways from the KEGG database confirmed a decreased enrichment of lipid storage, transport via lipoprotein, and upstream PPAR signaling pathway in placentas from PCOS‐mice compared with controls (Figure 2f). These results indicate a high turnover of acyl‐CoA and subsequent lipogenesis by fatty acid oxidation in response to androgen exposure in the placenta. Furthermore, the decrease in lipoprotein components suggests that the placenta with androgen exposure has a lower capacity to store and transport lipids.

By performing a co‐expression network analysis of all differentially expressed transcription factors and the differential expressions of their target genes, oestrogen‐related receptor beta (*Esrrb)* was identified to play central role in transcriptional regulation of the PCOS placenta. *Esrrb* expression was downregulated in the placentas of PCOS mice, together with several other transcription factors (Figure 2g). Consistent with previous findings that *Esrrb* functions as an important transcription factor for trophoblast lineage maintenance,^[^ ^32^ ^]^ the reduction of *Esrrb* in PCOS‐placentas may lead to altered trophoblast lineage proportions. Overexpansion of trophoblast giant cells and impaired labyrinth zone formation has previously been observed in *Esrrb* knockout mice,^[^ ^33^ ^]^ which aligned with our morphological observation of the PCOS placentas. To find a link between placental expression and embryonic development, we also performed an enrichment analysis with the mutations and quantitative trait loci leading to developmental phenotypes registered in the Mammalian Phenotype Ontology.^[^ ^34^ ^]^ The loss of function of *Esrrb* also leads abnormal brain development (Tables S5 and S6, Supporting Information). Considering that maternal hyperandrogenism has been linked to psychiatric disorders in offspring,^[^ ^35^ ^]^ *Esrrb* as an important player in bridging the hyperandrogenic in utero environment to abnormal placental and fetal development.

To clarify whether the observed expression changes are all ascribed to the known genetic risk variants for PCOS, we examined the expression levels of the genetic variants^[^ ^36^, ^37^ ^]^ in PCOS‐ and control‐placenta. PCOS is known to have a strong heritability and there are currently over 20 genetic variants identified to play a role in PCOS pathogenesis,^[^ ^36^, ^37^ ^]^ therefore we also investigated if any of the genes close to the PCOS‐associated genetic variants were perturbed at the expression level in mouse placenta exposed to hyperandrogenism. Among the gene regions that harbour the genome‐wide significant loci, high‐mobility group AT‐hook 2 *(Hmga2)* and Nei Like DNA Glycosylase 2 *(Neil2)* were downregulated at the mRNA levels in the PCOS‐placenta, which was prevented by flutamide cotreatment (Figure S2d, Supporting Information). As demonstrated in human placentas, the *HMGA2* expression is crucial to trophoblast invasion in early pregnancy.^[^ ^38^ ^]^ Moreover, of the genes significantly associated with PCOS in a transcriptome‐wide association study of 48 postmortem tissues,^[^ ^39^ ^]^ only four were expressed in our mouse PCOS placenta, and they are not differentially expressed (Figure S2d, Supporting Information). This suggests that expression regulation in the placenta is a rather exclusive endophenotype of PCOS.

### 2.4 Androgen Exposure Impairs Trophoblast Proliferation and Differentiation in the Placenta

**Figure 3:**
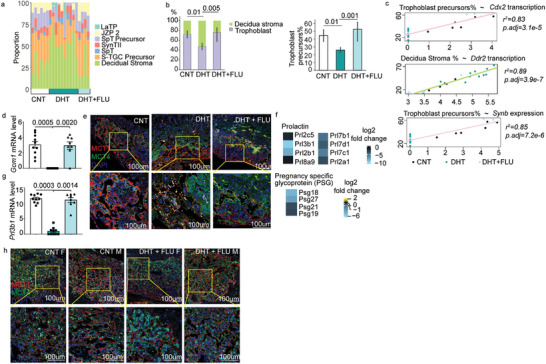
Flutamide treatment prevented the impaired placenta development induced by maternal androgen exposure. a) Cell type proportions deconvoluted by CIBERSORTx. b) Bar plots representing mean proportion of trophoblast cells and trophoblast precursors. The error bars indicate 95% confidence intervals. P values by ANCOVA to control for litter. c) linear regression of selected DEGs and trophoblast or trophoblast precursors proportion. P values are adjusted using the Holm method. d) *Gcm1* mRNA levels from RNA sequencing at E10.5. P values calculated by ANCOVA to control for litter. e) Representative confocal microscopy images of E10.5 mouse placenta stained for syncytiotrophoblast layer I and II (SynTI and SynTII) respectively with MCT1, MCT4, and DAPI; upper row images were at 10X magnification and lower row at 20X magnification. f) Gene expression level of prolactin and pregnancy specific glycoproteins (PSGs) from RNA sequencing at E10.5. g) *Prl3b1* expression from RNA sequencing at E10.5. h) Representative confocal microscopy images of E13.5 mouse placenta stained for SynTI and SynTII respectively with MCT1, MCT4, and DAPI; upper row images were at 10X magnification and lower row at 20X magnification. Number of dams used for RNA sequencing analysis: For embryonic stage E10.5, CNT = 3, DHT = 1, DHT+FLU = 3. For embryonic stage E13.5, CNT = 3, DHT+FLU = 3.

From E10.5, the mouse placenta undergoes a significant transformation, marking the shift from the primitive choriovitelline configuration to the chorioallantoic configuration. This transformation involves the expansion of the embryonic labyrinth and the progressive differentiation of trophoblast cell lineages, which are tightly regulated by different transcription factors, for example, *Esrrb*.^[^ ^30^ ^]^ To understand the impact of maternal androgen excess on placental development and cell type composition, we deconvoluted our bulk RNA‐seq expression data using the cell type‐specific annotation from single‐nuclei data at E10.5.^[^ ^40^ ^]^ The placentas from PCOS‐mice exhibited an increased proportion of decidua stromal cells but a decreased proportion of trophoblast cells with nearly 50% less trophoblast precursor cells compared to the control and flutamide‐treatment (**Figure** **3**a,b; Table S7, Supporting Information).

The reduction of trophoblast precursors may be caused by the drastic decrease in the expression of stem cell maintenance genes, such as *Cdx2*, which positively correlated with the percentage of trophoblast precursors that was significantly reduced in PCOS‐placenta (Figure 3b,c). The gene expression of *Ddr2*, encoding the discoidin domain receptor tyrosine kinase 2 and responsible for sensing the extracellular matrix (ECM) collagens,^[^ ^40^ ^]^ was positively correlated with decidua stroma which was increased in the PCOS‐mice placenta (Figure 3c). Indeed, the ECM including collagens, was increased in the PCOS‐mice placenta (Figure 2b,d), which could impede the proper proliferation and invasion of trophoblasts.^[^ ^41^ ^]^ The PCOS‐mice placenta retained more LaTP (labyrinth trophoblast precursor) cells instead of differentiating into SynTI (syncytiotrophoblast layer I) and SynTII (syncytiotrophoblast layer II) branches (Figure S2e, Supporting Information). Correlation of gene expressions with the proportions of cell types showed that *Synb* expression was positively correlated with trophoblast progenitor cells (Figure 3c). And the expression of *Aqp3* (Aquaporin 3) correlated strongly with the LaTP cells arrest (Figure S2f, Supporting Information). The *Aqp3* plays a role in the transport of glycerol, free fatty acids, and triglycerides and subsequently influence the fetal growth in mice.^[^ ^41^ ^]^ These findings suggest that excess maternal androgens cause dysregulation of the proliferation and differentiation of trophoblast precursor cells, consequently impair trophoblast fusion.

To validate the cell type‐specific defects, immunofluorescent staining of trophoblast precursor cells and syncytiotrophoblast cells was performed on E10.5 and E13.5 placentas. In mouse placentas, syncytiotrophoblast precursors undergo terminal differentiation before fusing to form two layers of syncytium: Syn I and Syn II. This process is regulated by glial cells missing transcription factor 1 (*Gcm1*), syncytin A (*SynA*)^[^ ^42^ ^]^ and syncytin B (*SynB*).^[^ ^43^, ^44^ ^]^ Maternal androgen exposure resulted in depletion of *Gcm1* (Figure 3d) and *SynB* (Figure S2g, Supporting Information), leading to substantial reduction in the formation of Syn I and Syn II layers in E10.5 placenta (Figure 3e). Consequently, a decrease in the expression of gene families of prolactin and pregnancy‐specific glycoproteins (PSGs) in PCOS‐mice placenta was observed, as these are secreted by trophoblast (Figure 3f). In addition, the labyrinth zone patterning in PCOS‐mice placentas was altered, with increased trophoblast giant cells (TGCs) population compared to control placentas (Figure 3f).

Although morphologically there was increased presence of trophoblast giant cells in the PCOS‐mice placentas at E10.5, the proportion of sinusoidal‐TGCs was lower (Figure 3a), along with a reduction in the sinusoidal‐TGC markers *Prl3b1* and *Ctsq* (Figure 3g, Figure S2g, Supporting Information). In line with placenta transcriptome analysis, flutamide treatment effectively prevented the aforementioned placental abnormalities (Figure 3d,e and 3g). PCOS‐mice showed embryonic lethality before E13.5, possibly attributed to the deformation of the labyrinth zone and dysregulation of critical genes in placental development.^[^ ^45^, ^46^ ^]^ Further analyses of the formation of the placental labyrinth zone, especially Syn I and Syn II layers at E13.5, revealed that placentas from flutamide‐treated PCOS‐mice closely resembled those of controls (Figure 3h).

Interestingly, when comparing DEGs at E10.5 to their expected regulation during normal pregnancy progression at E13.5, we found that most of the DEGs at E10.5 were regulated in the opposite direction at E13.5 (Figure S2h, Supporting Information). Specifically, genes involved in the differentiation from LaTP to SynII cells in the labyrinth and the invasive sinusoidal‐TGC were down‐regulated in PCOS‐mice, contrary to the expected to be up‐regulation at E13.5 (Figure S2i, Supporting Information), indicating that maternal androgen exposure impedes the labyrinth differentiation.

### 2.5 Flutamide Treatment Prevented the Methylation Induced by Maternal Androgen Exposure

**Figure 4:**
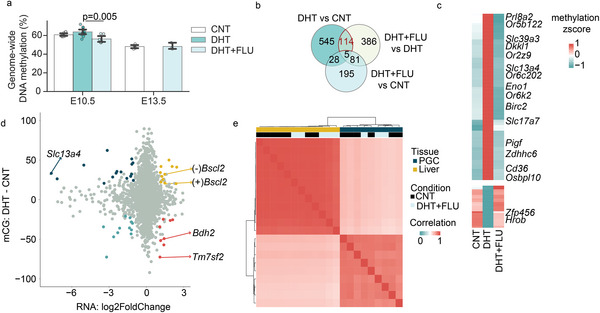
Flutamide co‐treatment protects against DNA methylation disturbances in placenta induced by androgen exposure while maintaining the DNA transcription in PGC, embryonic, and offspring liver tissues. a) Bar plots representing 5mC (5‐methylcytosine) level. The error bars indicate 95% confidence intervals. P values by ANCOVA to control for litter. b) Veen diagram showing the intersection of the genes annotated with the differentially methylated promoters in each comparison. c) The methylation z‐score ((methylation level of each sample – mean value of methylation levels)/SD of methylation levels) of the genes whose promoter methylation was dysregulated by 10 mm DHT pellet insertion and reversed by flutamide cotreatment. d) In placenta at E10.5, the differentially expressed genes by DHT treatment and the 5mC sites methylation difference on their promoters. The genes with differentially methylated sites (FDR<5%, difference>20) were colored. e) Correlation heatmap of the gene expression in E13.5 PGC and embryonic liver. Number of dams used for WGBS analysis: For embryonic stage E10.5, CNT = 3, DHT = 1, DHT+FLU = 3. For embryonic stage E13.5, CNT = 3, DHT+FLU = 3.

The methylation of promoter regions has been associated with alterations in gene transcription,^[^ ^47^ ^]^ and plays a crucial role in the formation of different trophoblast lineages.^[^ ^7^ ^]^ Therefore the placental methylation profile was investigated. The establishment and maintenance of global DNA methylation were only marginally increased by androgen exposure at E10.5 (**Figure** **4**a). To specifically examine the 5mC (5‐methylcytosine) sites in gene promoters, we conducted genome‐wide DNA methylation profiling to identify androgen exposure‐associated 5mC methylation alterations. At E10.5, androgen exposure led to a slight increase in global 5mC levels in the placenta (ANCOVA, P = 0.25), while flutamide placenta maintained the 5mC level at both E10.5 and E13.5 similar to that of control placenta (ANCOVA, P_CNT versus DHT + FLU_ = 0.11) (Figure 4a).

By quantifying the number of genes with promoters exhibiting distinct 5mC sites between groups, we identified 692 genes associated with PCOS‐mice compared with controls, 586 genes associated with PCOS‐mice compared with flutamide‐mice, and only 309 in flutamide‐mice compared with controls (FDR < 5%, methylation difference > 20%) (Figure 4b). The differentially methylated 5mC sites were mainly located within CpG shores, known to be susceptible to methylation changes in tissue specification and certain disease progress (Figure S2j, Supporting Information).^[^ ^48^, ^49^ ^]^ Moreover, the androgen‐exposed placentas also affected methylated sites in the promoter region. In the placentas from the flutamide‐mice, the 5mC methylation changes on the promoters of 114 genes were reversed (Figure 4c). Among them, *Slc13a4* was hypermethylated in the promoter of PCOS‐mice but remained unaffected in flutamide‐mice (Figure 4d). This hypermethylation might contribute to the sevenfold downregulation of *Slc13a4* transcription in PCOS‐mice placenta (Figure 4d). As *Slc13a4* encodes the most abundant sulfate transporter located in the syncytiotrophoblast, the *Slc13a4* loss‐of‐function in mouse placentas led to fetal death.^[^ ^50^ ^]^ These findings suggest that the mid‐gestation lethality in PCOS‐mice resulted from both transcriptional and epigenetic aberrations.

Despite flutamide preventing adverse effects on both placenta and embryo development, concerns arise regarding its long‐term effects on offspring health, especially the potential hepatotoxicity. In the E13.5 embryonic liver, there were no differentially expressed genes between the control and flutamide‐treated groups (Figure 4e). To investigate the potential offspring health, PGCs were assessed as germ cells are essential in transmitting parental information to the offspring in mice. At E10.5, we collected migrating PGCs for RNA sequencing and DNA methylation profiling. Despite a limited number of samples, the transcriptome of PGCs from embryos of flutamide‐treatment was comparable to that of controls (Figure S2k, Supporting Information). Based on the DNA methylation of all 5mC sites, PGCs from embryos of flutamide‐treated mice clustered with those of controls but were separated from the PCOS‐mice (Figure S2l, Supporting Information). At E13.5, the gene expression profile of germ cells remained similar in flutamide‐mice compared to controls (correlation > 0.94, Figure 4e).

### 2.6 Androgen Exposure Affects Villous Cytotrophoblast Differentiation in Human Trophoblast Organoids

After observing a DHT‐induced reduction of trophoblast differentiation capacity in the mouse placenta, we decided to further explore the translatability of these findings to humans. Therefore, human trophoblast organoids (TOs) were established using human trophoblast stem cells. The organoid consists of a stem‐like cytotrophoblast layer and differentiated syncytiotrophoblast layers. To further explore how DHT may affect the differentiation capacity of human trophoblasts, human TOs were induced for differentiation with DHT supplementation. The invasive trophoblast cell type, extravillous cytotrophoblast, are formed upon differentiation, which is critical for the successful establishment of pregnancy. Under in vitro conditions, the differentiated extravillous cytotrophoblasts migrate through the Matrigel, leaving a visible trace for observation.^[^ ^51^ ^]^ In addition, we also included a group with combined DHT and flutamide treatment, mimicking our experimental setup in the mouse experiments. As a result, our in vitro experiment set up included the control group (CNT), the group with 1 nm DHT (DHT), and the group with 1 nm DHT + 2um flutamide co‐treatment (DHT + FLU).

**Figure 5:**
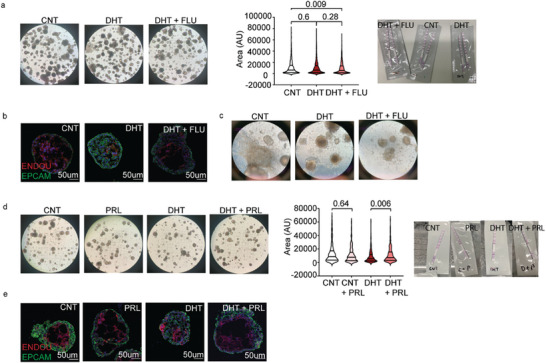
Effect of DHT on human placenta trophoblast organoids is prevented by flutamide treatment. a) Representative images of human trophoblast organoids (TOs) grown in matrigel upon treatment of DHT and/or flutamide, images were taken at 4X magnification. And size measurement of TOs and detection of human chorionic gonadotrophin (hCG) production with commercial pregnancy tests. b) Representative confocal microscopy images of organoids stained for cytotrophoblast and syncytiotrophoblast with EPCAM, ENDOU, and DAPI, images were taken at 20X magnification. c) Representative images of human placenta organoids grown in matrigel after 10 days of differentiation treatment, images were taken at 10X magnification. d) Representative images of human placenta organoids grown in matrigel upon treatment of DHT and/or prolactin, images were taken at 4X magnification. And size measurement of TOs and detection of hCG production with commercial pregnancy tests. e) Representative confocal microscopy images of organoids stained for cytotrophoblast and syncytiotrophoblast with EPCAM, ENDOU, and DAPI, images were taken at 20X magnification. TO size measurement: Kruskal‐Wallis test with Dunn's post hoc test due to data sets being non‐parametric.

No discernible difference in the appearance, morphology, or size of TOs was observed between CNT, DHT, or DHT + FLU groups (Kruskal‐wallis test, *H* = 8.884; *P* = 0.012), and all groups produced human chorionic gonadotrophin (hCG) to a level detectable by commercial pregnancy tests (**Figure** **5**a). Immunofluorescent staining of cytotrophoblast and syncytiotrophoblast markers using EPCAM and ENDOU, respectively revealed that both the CNT and DHT + FLU groups exhibited a typical TO structure. However, TOs treated with DHT had less syncytiotrophoblast and an increased number of cytotrophoblast cells in the center of the TOs (Figure 5b). Upon induction of differentiation, both CNT and DHT + FLU TOs showed extensive migration of extravillous trophoblasts into the matrigel, while less migration was observed in TOs treated with DHT (Figure 5c). These results validated our findings in the mouse placentas and suggested that androgen exposure profoundly influenced the differentiation of human trophoblast precursors and the invasion of trophoblast cells.

Considering the severe downregulation of several genes in the prolactin family in E10.5 PCOS‐mice placenta (Figure 3f), we next explored whether prolactin supplement could prevent the adverse effects induced by androgen exposure, given its previously demonstrated role in enhancing differentiation and invasion of human trophoblast cells.^[^ ^52^ ^]^ The prolactin experiment consisted of the following groups: control (CNT group), 100 ng mL^−1^ prolactin (PRL group), 1 nm DHT (DHT group), 1 nm DHT + 100 ng mL^−1^ prolactin (DHT + PRL group). Prolactin did not alter the overall morphology and hCG production in TOs, with or without DHT. However, TO size was increased in the DHT + PRL group compared to the DHT‐only group (Kruskal‐wallis test, *H* = 22.22; *P* < 0.0001) (Figure 5d). Immunofluorescence staining showed that prolactin treatment improved syncytiotrophoblast differentiation in the DHT + PRL group compared to the DHT group (Figure 5e) without affecting the normal trophoblast differentiation in PRL group. Collectively, these findings suggest that PRL had the potential to promote cytotrophoblast differentiation in placentas under PCOS condition.

## 3 Discussion

Women with PCOS are at increased risk of pregnancy complications, and their progeny are predisposed to an altered growth trajectory and an elevated likelihood of acquiring a PCOS diagnosis, along with associated comorbidities during adulthood.^[^ ^53^ ^]^ The precise mechanism mediating the transmission of the disease via the maternal‐fetal environment remains unexplored. Using the peripubertal DHT‐exposure model,^[^ ^53^ ^]^ we could analyze a hyperandrogenic maternal environment throughout the entire gestation mimicking human PCOS pregnancy. Specifically, we sought to determine whether continuous maternal androgen exposure‐induced alterations could be mitigated through targeted treatment of the androgen receptor, utilizing flutamide. Indeed, the induction of a classic PCOS‐like reproductive and metabolic phenotype in F0 dams was successfully counteracted by blocking the androgen receptor pathway, in line with previous research.^[^ ^17^, ^18^ ^]^ Moreover, in utero hyperandrogenism resulted in a global reduction of the transcriptional activity in the placentas leading to diminished placental labyrinth formation as well as impaired proliferation and differentiation of trophoblast precursors. Consequently, syncytialization was severely compromised, potentially contributing to defective nutrient transport and embryonic lethality at mid‐gestation. These deleterious effects were completely prevented by inhibition of the androgen receptor pathway with flutamide, resulting in fetal development comparable to controls. Analogous findings were observed in the human trophoblast organoid experiment, indicating conserved effects of hyperandrogenism in disrupting maternal‐fetal interaction.

The maintenance of trophoblast stem cell population is critical for supporting its differentiation into different trophoblast cell lineages supporting placenta function.^[^ ^54^ ^]^ The markedly reduced trophoblast precursors at E10.5 in PCOS‐mouse placentas are consistent with previous findings with reduced proliferation of cytotrophoblasts in women with PCOS.^[^ ^55^ ^]^ This observed reduction might be attributed to a significant downregulation of genes that contribute to maintaining trophoblast stem cells, including *Esrrb*, *Cdx2*, and *Elf5*. Notably, the deletion of either *Elf5* or *Esrrb* causes embryonic lethal during early or mid‐gestation in mice.^[^ ^56^, ^57^ ^]^ Therefore, these genes could be the driver of the embryonic lethality and placental dysfunction observed in PCOS‐mice.

One vital step of pregnancy establishment in species with the hemochorial placenta involves the proliferation and differentiation of trophoblast stem cells together with the invasion of trophoblasts into the maternal decidua for the remodeling of spiral arteries, a process orchestrated by trophoblast giant cells (TGCs) in mice and extravillous trophoblasts in humans.^[^ ^8^, ^58^ ^]^ The formation of syncytiotrophoblast and sinusoidal‐TGCs, two important cellular components of the labyrinth zone was severely compromised under maternal hyperandrogenic environment. Sinusoidal‐TGCs separate the maternal sinusoids and syncytiotrophoblast layers and are in direct contact with maternal blood circulation facilitating maternal‐fetal connection and nutrient transfer. Indeed, genetic ablation of sinusoidal‐TGCs is associated with intrauterine growth restriction and embryo death at late gestation.^[^ ^59^ ^]^ The great decrease in sinusoidal‐TGC marker, *Ctsq*, expression in placentas coincides with the miscarriage in PCOS‐mice.

Furthermore, the finding that androgen exposure impedes the invasion of extravillous trophoblasts was confirmed in human trophoblast organoids, suggesting a potential link to miscarriage in the PCOS‐mice and women with PCOS. Trophoblast giant cells, known for their endocrine functions, secrete prolactin in mouse placentas. Prolactin is an important hormone involved in angiogenesis and the regulation of immune responses. The substantial downregulation of nine members of the prolactin family, with expression decreases ranging from 1–10‐fold in androgen‐exposed mouse placentas, may have detrimental effects on embryo survival, leading to observed mid‐gestation embryo lethality. While the role of prolactin in the human placenta is less explored, it has been suggested to improve trophoblast migration and invasion and to decrease placental inflammation.^[^ ^52^, ^60^ ^]^ Consistent with previous findings, our observations revealed improved cytotrophoblast differentiation into syncytiotrophoblast in human trophoblast organoids under a hyperandrogenic environment when supplemented with prolactin, implying a protective role of prolactin in maternal‐fetal interaction.

Another critical aspect of pregnancy maintenance involves nutrient and gas transport via the placenta, which is mediated via syncytiotrophoblasts in both mice and humans. The differentiation of syncytiotrophoblasts from trophoblast stem cells requires *Gcm1* and syncytin in mice and humans. *Gcm1* promotes trophoblast stem cells to syncytiotrophoblasts and extravillous trophoblasts differentiation,^[^ ^61^ ^]^ while syncytin induces cell‐cell fusion for the formation of syncytiotrophoblast layers.^[^ ^43^, ^44^ ^]^ The 6‐to‐7‐fold decreased expression in both genes contributed to disruption of trophoblast stem cells to syncytiotrophoblasts differentiation thus malformation of syncytiotrophoblast layers. To date, no studies have investigated the impact of PCOS on syncytiotrophoblasts in the human placenta, although a reduced number of syncytiotrophoblasts has been associated with in utero growth restriction and pre‐eclampsia, both of which are observed in PCOS pregnancies.^[^ ^62^, ^63^ ^]^ We showed that DHT exposure impaired syncytiotrophoblast differentiation in human trophoblast organoids, confirming the role of impaired syncytiotrophoblast lineage in PCOS pregnancies.

Fatty acid uptake in trophoblast cells is facilitated by Acsl‐mediated conversion into acyl‐CoA, which is used for subsequent metabolic processes like β‐oxidation.^[^ ^64^ ^]^ Although maternal androgen exposure increases placental fatty acid metabolism and acyl‐CoA uptake, there was a concurrent increase in *Nudt7* and *Hmgcs2* expression. This may lead to diminished levels of CoA derivatives in the placenta, thereby inducing mitochondrial stress, inflammation, and pre‐eclampsia.^[^ ^65^ ^]^ Additionally, diminished FABP (fatty acid binding protein), PPARɤ and its functional unit RXRɑ expressions were all observed in the PCOS placenta. FABPs bind fatty acids in the cytoplasm and transport them to their final destinations, including to the nuclear receptors, PPAR, and RXR. Considering that the activation of PPARɤ‐RXR heterodimer positively regulates fatty acid uptake in human trophoblasts,^[^ ^66^ ^]^ we hypothesized that maternal hyperandrogenism results in restricted fatty acids transport at the maternal‐fetal interface. This limitation could lead to a constrained energy supply for fetal development, potentially resulting in embryo lethality.

Consistent with previous findings indicating that flutamide restores the endometrium receptivity,^[^ ^17^ ^]^ our study reveals comparable placental and liver transcriptional profiles between control mice and those treated with flutamide in the PCOS model, suggesting the prevention of hyperandrogenism‐induced adverse events. However, it is noteworthy that flutamide treatment during pregnancy potentially impedes male reproductive system development.^[^ ^67^ ^]^ Therefore, in women with PCOS, flutamide use is normally combined with other first‐line treatment options to treat hirsutism related symptoms only,^[^ ^68^ ^]^ and it should not be used during human pregnancy.^[^ ^69^ ^]^

## 4 Conclusion

**Figure 6:**
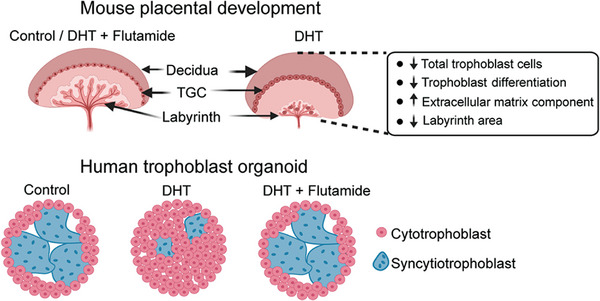
Summary of the key findings. Maternal hyperandrogenism leads to the formation of smaller labyrinth zone with dysregulated trophoblast giant cell (TGC) formation in the mouse placenta. This might be attributed to reduced total trophoblast cells, impaired trophoblast differentiation capacity, and increased extracellular matrix upon maternal androgen exposure. This impaired trophoblast differentiation by androgen exposure is recapitulated by human trophoblast organoid treated with DHT, resulting in reduced syncytiotrophoblast formation. Interestingly, flutamide can prevent unfavorable events induced by androgen both in mouse placenta and human trophoblast organoids.

All together, we revealed mid‐gestation embryo lethality and deleterious reprogramming of placental development in the androgenized mouse PCOS model. Notably, comparable adverse effects were observed in human trophoblast organoids, suggesting the presence of conserved effects induced by hyperandrogenism. All identified detrimental effects were effectively prevented by blocking androgen receptors, highlighting the crucial role of the androgen‐mediated pathways in the pathogenesis of PCOS placentas (**Figure** **6**). These findings hold promise for future research aimed at discovering safe androgen receptor blockers and related strategies to mitigate the adverse impact of hyperandrogenism on pregnancy outcomes and offspring health.

## 5 Experimental Section

### Ethics Approval Statement

All animal experiments were approved by Swedish board of Agriculture (Jordbruksverket, ethical approval number: DNR20485‐2020). Animal care and procedures were performed in accordance with guidelines specified by European Council Directive and controlled by Comparative Medicine Biomedicum (KM‐B), Karolinska Institutet, Stockholm, Sweden. The use of human first trimester placenta tissues to derive trophoblast stem cells was approved by the Medical University of Vienna ethics boards (no. 084/2009).

### Animals and Treatment

3‐week‐old C57Bl/6J female mice were purchased from Janvier Labs (Le Genest‐Saint‐Isle, France). Mice were housed five per cage in IVC‐GM 500 cages with maintained temperature (22 °C) and humidity (55–65%) at a 12/12 h light/dark cycle and fed with in house chow diet. DHT pellets were prepared according to previously published protocol.^[^ ^26^, ^27^, ^70^, ^71^ ^]^ Briefly, Dow Corning Silastic tubing 0.04 mm inner diameter × 0.085 mm outer diameter (Fisher Scientific, Hampton, NH) was filled with DHT (≈5.24 mg for 10 mm DHT pellet, 5α‐androstan‐17β‐ol‐3‐one; A8380, Sigma‐Aldrich), and 2 mm medical adhesive silicone (Factor II, Lakeside, AZ) was used to seal the tube on both sides. For control pellets, 14 mm empty pellets were sealed directly. Pellets were incubated in saline for 24 h at 37 °C for equilibration before insertion.

At 4 weeks of age, mice were randomly divided into 3 groups: control, DHT (i.e., PCOS), and DHT with flutamide (DHT + FLU), i.e., PCOS and flutamide. In the PCOS group, a DHT pellet was implanted, and the control group received an empty pellet under light isoflurane anesthesia. Co‐treatment with flutamide was done by implantation of a 90‐day continuous‐release pellet containing 25 mg flutamide (Innovative Research of America, Sarasota, FL, USA) at the same time as the DHT pellet.

### Superovulation and Mating

Four weeks after pellet implantation, mice from all groups were superovulated with 5IU pregnant mare's serum gonadotropin (PMSG; Folligon, MSD Animal Health Care) followed by 5IU human chorionic gonadotropin (hCG; Pregnyl 5000IE, Merck Sharp & Dohme AB, Stockholm, Sweden) 48 hours later. Females were then mated with wild type, unexposed males overnight right after hCG injection, and the morning after mating was counted as embryonic day (E) 0.5. At embryonic day E10.5 and E13.5 pregnant females were dissected, and pregnancy was defined as >2 g increase in body weight from E0.5.

### Reproductive Phenotypic Assessments

A separate batch of F_0_ mice was kept until 10–13 weeks of age, i.e., 7 weeks after pellet implantation, when their reproductive phenotypes were assessed. Estrus cyclicity was assessed by vaginal smear and cytology for 12 consecutive days as previously described.^[^ ^6^ ^]^ The AGD was assessed in F_0_ mice at the time of dissection.

### Metabolic Phenotype Assessments

The body weight of F_0_ mice was monitored throughout the experiment (from pellet implantation to 10 weeks after the treatment started).

### Tissue Collection

At the end of phenotypic assessments, mice were subjected to tissue collection. Two hours fasting was performed before dissection, and tissue weight was recorded after collection. Tissues were snap frozen directly after collection in liquid nitrogen and stored in −80 °C. The serum hormone measurement was performed with LC‐MS/MS method as previously described.^[^ ^72^ ^]^

### Histological Analysis of the Placenta

E10.5 and E13.5 placenta were dissected and fixed in 4% PFA overnight, dehydrated, and embedded in paraffin. The entire placenta was sectioned at 5 µm thickness and the sections in the middle were used for staining and histological analysis. The sections were subjected for haematoxylin & eosin staining or stored at room temperature for later immunofluorescence (IF). One or two representative images were taken per section with a light microscope at × 10 magnification (Zeiss Axioplan). The images were corrected for white balance with ImageJ (version 1.52 h) and different layers were labeled for illustration.

### Immunofluorescence (IF)

*Placenta*: For IF staining, placenta sections from the middle of the tissue containing all three structural layers were deparaffinized and rehydrated, then antigen retrieval was performed with citrate buffer (C9999, Sigma‐Aldrich) for 6 mins in microwave (800 W). After cooling down, sections were permeabilized with 0.1% Tween 20 then blocked with 5% donkey serum in PBS for 1 hour at room temperature. Then the sections were incubated overnight at 4 °C with primary antibody (MCT1‐Sigma ab1286‐I, MCT4‐Sigma ab3314P). The next day, sections were washed and incubated with secondary antibody (donkey anti‐chicken IgG 647, Invitrogen; donkey anti‐rabbit IgG 594, Invitrogen). Sections were then washed and incubated with DAPI then mounted with anti‐fade mounting medium with DAPI (H‐1800, Vectashield).

*Human Trophoblast Organoid*: Human trophoblast organoids were collected and fixed in 4% PFA overnight, they were then proceeded for dehydration with 30% sucrose (57 501, Merck) for another 24 hours. Organoids were then washed and embedded in OCT for cryo‐sectioning on Epredia NX70 cryostat. Sections are at 5 µm thickness and stored in −80 °C until use. At the time of IF staining, sections were let to sit at room temperature for 10 min before start, then they were washed twice with PBS followed by blocking with PBST (5% BSA and 0.3% Triton‐100 in PBS) for 2 h at RT. Primary antibody (anti‐ENDOU, HPA012388 – for syncytiotrophoblast labeling, Sigma‐Aldrich; anti‐EpCAM, VU1D9, Cell Signaling Technology – for cytotrophoblast labeling) incubation was done overnight at 4 °C followed by washing and 1 hour of secondary antibody (donkey anti‐mouse IgG 488, Invitrogen; donkey anti‐rabbit IgG 594, Invitrogen) incubation at RT. Sections were then washed and incubated with DAPI then mounted with anti‐fade mounting medium with DAPI (H‐1800, Vectashield). Confocal images were taken with Zeiss LSM800 confocal microscopy at 10X, 20X, or 40X depending on the sample type.

### Embryo Dissection and Isolation of Primordial Germ Cells

The placenta and embryos were dissected at E10.5 and E13.5 in ice‐cold 1x PBS using a dissection microscope (VWR). At each time point, the fetal crown–rump length and weight were measured after dissection, and embryos and placenta were imaged at 1.5x (E10.5) and 1x (E13.5) magnification. Embryos were subjected to PGC isolation and/or staining of PGCs using immunohistochemistry (IHC). For IHC: At E10.5, two whole embryos per dam were fixed and incubated with Anti‐STELLA antibody to investigate germ cell migration by whole mount staining (see below) and images were acquired using a confocal laser‐scanning microscope. For E13.5 embryos, the gonads from 1 male and 1 female embryo per dam were collected and fixed in 4% PFA overnight, then subjected to IHC for PGC counting. For PGC isolation, the central body containing the hindgut of the remaining E10.5 embryos was dissected, dissociated and FACS sorted to separate somatic and germ cells using SSEA‐1 antibody and the integrin beta 1 (CD61) for WGB and RNA sequencing (see below).^[^ ^73^ ^]^ The gonads from each E13.5 embryos were dissected and sex determinate before they were pooled together based on sex and dam for FACS sorting and WGB and RNA sequencing (see below).^[^ ^73^ ^]^

*Whole mount staining* of the E10.5 embryos was performed according to previously established protocols with modification.^[^ ^74^ ^]^ Briefly, three E10.5 embryos from each group were fixed in 4% PFA on rotation at 4 °C for 2 h with rotation, followed by rising with TPBS (PBS + 0.3% Triton‐X) at 4 °C for three times at 1 h interval. Then the embryos were incubated with rabbit anti‐STELLA (1:1000, Abcam) in TPBS for at 4 °C overnight with rotation. This was followed by rinsing the samples overnight in TPBS at 4 °C with rotation. On the next day, the samples were incubated with Donkey anti‐rabbit IgG conjugated with Alexa488 in TPBS (1:2000, Life Technologies) overnight at 4 °C with rotation. Samples were then washed in TPBS overnight at 4 °C with rotation and stored in dark at 4 °C until the clearing process. Tissue clearing was performed by washing the samples at room temperature sequentially in 50% Methanol/PBS for 5 mins with rotation, 3 times in 100% Methanol for 20 min, and lastly in BABB (benzyl alcohol/benzyl benzoate; Sigma Aldrich) for 5 mins. Samples were further cleared in BABB for ≈1 h at RT with rotation until it became completely transparent. Cleared samples were immediately proceeded for confocal microscopy (Zeiss LSM880).

*Microscope*: Whole mount cleared embryos were transferred to glass bottom dishes (MatTec) containing BABB for imaging with Zeiss LSM880 confocal microscope.

*Image Processing*: For whole mount‐stained embryos, the stacked images were first converted to imaris file using ImarisFileConverter and analyzed using Imaris x64 software (version 9.5.1, Bitplane). The surface tool and mask function were used to crop out region of PGC in the whole embryo. Then the spot function was applied to the cropped region to automatically identify PGCs. Identified PGCs were manually checked to insure correct identification. The number of PGCs for each embryo was used for further analysis.

*Dissociation and FACS Sorting*: The specimens for FACS were dissociated in dissociation buffer containing 1 mg mL^−1^ Collagenase (Sigma C0130) + 0.1 mg mL^−1^ DNaseI (Roche 11 284 932 001) in PBS at 37 °C for 6 min with slow shaking. After that the specimens were further dissociated by pipetting in washing buffer containing 0.1% BSA (Sigma A7030) + 0.1 mg mL^−1^ DnaseI in PBS on ice. Then the cell suspension was centrifuged at 220 g for 5 min and the cell pellets rinsed twice with washing buffer. The resulting pellets were resuspended in 200 µL of chilled washing buffer and incubated with PE‐conjugated anti‐CD61 antibody (1:200, BioLegend 104 307) and eFluor660‐conjugated anti‐SSEA1 antibody (1:20, eBioscience 50‐8813‐42) for 15–30 min on ice. After that, the cells were rinsed twice with washing buffer. Prior to cell sorting, the cell suspension was further dissociated by passing through a 35um cell strainer (Corning 352 235) and stained with cap Fixable viability Dye fluor450 (eBiscience 65‐0863‐14) or Dapi. FACS sorting was performed with a 100 mm nozzle using a flow cytometer (SONY SH800S) following the manufacturer's instructions. Embryonic germ cells (SSEA+CD61+) and somatic cells (SSEA‐CD61‐) were sorted directly into Smartseq3 or whole genome bisulphite lysis buffer for downstream ​processing for RNA sequencing and whole genome bisulphite sequencing and stored at −80 °C until use.

### Human Trophoblast Organoid Generation

The human trophoblast stem cell lines were generously given by our collaborator Sandra Haider. The trophoblast stem cells were isolated from 1^st^ trimester placenta, after the isolation and culture process villous cytotrophoblasts were left in the culture, giving rise to the trophoblast stem cells. They were cultured and developed into organoids according to previous publications.^[^ ^51^ ^]^ Briefly, trophoblast stem cells were cultured in Fibronectin (Sigma‐Aldrich FC010) coated plate, and the culture medium was Advanced DMEM/F12 (Gibco 11 540 446) supplemented with 1x B‐27 Supplement (50X) minus vitamin A (Gibco 12 587 010), 1x Insulin Transferrin Selenium Ethanolamine (ITS‐X) (Gibco 10 524 233), 1x L‐glutamine(Thermo Fisher 25 030 024), 1 µm A83‐01 (Tocris 2939), 3 µm CHIR99021 (Tocris 4423), 50 ng mL^−1^ hEGF (Gibco PHG0311), 5 µm Y‐27632 (Sigma Aldrich Y0503), 0.1 mg mL^−1^ Gentamicin (Fisher Scientific 15‐710‐064) and 0.01 m HEPES (Gibco 15710‐049). Once the trophoblast stem cells reach 80–90% confluency, they were seeded into Matrigel to form trophoblast organoids. Detailed procedure of organoid formation was performed according to previous publications. Briefly, 7–8000 total trophoblast stem cells were mixed with 20 µl of Matrigel and this Matrigel mix drop was placed onto a pre‐warmed 48‐well plate. The plate was put in the incubator for 15 minutes before the addition of 250 µL of trophoblast medium. The trophoblast medium composition was as follows: advanced DMEM/F12, 1x B27 without vitamin A, 1x ITS‐X, 1x L‐glutamine, 0.1 mg mL^−1^ Gentamicin, 0.01 m HEPES, 1um A83‐01, 3 µm CHIR99021, 100 ng mL^−1^ hEGF, 5 µm Y‐27632. For experimental purposes to mimic a hyperandrogenic environment, the following 2 mediums were prepared based on the trophoblast medium: DHT medium with the addition of 1 nm DHT and FLU medium with the addition of 1 nm DHT and 2 µM Flutamide. For the prolactin experiments, the addition of 100 ng mL^−1^ prolactin was added to the trophoblast medium with or without the supplementation of DHT. The medium was changed every 2–3 days until the majority of the organoids in control medium reach 200 mm in diameter.

*Differentiation of Human Trophoblast Organoid*: To assess the ability of trophoblast stem cells to differentiate into extravillous trophoblasts, we stimulated the differentiation with the following differentiation medium. Diff1: advanced DMEM/F12, 1x B27 without vitamin A, 1x ITS‐X, 1x L‐glutamine, 0.05 mg mL^−1^ Gentamicin, 0.01 m HEPES, 2uM A83‐01, 50 ng mL^−1^ hEGF. Diff2: advanced DMEM/F12, 1x B27 without vitamin A, 1x ITS‐X, 1x L‐glutamine, 0.05 mg mL^−1^ Gentamicin, 0.01 m HEPES, 50 ng mL^−1^ hEGF. When the majority of the organoids reach the size of 200 mm, Diff1 was used to stimulate differentiation for 5 days, after 5 days, the organoids were wash with washing medium (composition: advanced DMEM/F12, 1x B27 without vitamin A, 1x ITS‐X, 1x L‐glutamine, 0.05 mg mL^−1^ Gentamicin, 0.01 m HEPES) then supplemented with Diff2 medium for another 5 days before they were harvested for RNA extraction. For experimental purpose for mimicking the hyperandrgenism and study the effect of flutamide, the following differentiation medium was prepared: DHT Diff1 and DHT Diff2 mediums with the supplementation of 1 nm DHT, and Flu Diff1 and Flu Diff2 mediums with the supplementation of 1 nm DHT and 2um flutamide.

*hCG measures*: At the end of each experiment, hCG was measured in each plate with commercial pregnancy test strips (Gravidtetstest GI29100, Medistore) with a sensitivity of 20mIU mL^−1^ and reliability of 99.9%.

*Human Trophoblast Organoids Size Measurement*: At the end of experiments, pictures were taken for the organoids using microscope at 4X magnification with a scale. Images were processed with CellProfiler (version 4.0.7) for organoid identification and size measurement.

### Smart‐Seq3 Bulk RNA Sequencing Library preparation

cDNA libraries for placenta and PGCs were generated using the Smart‐seq3 protocol.^[^ ^75^ ^]^ Half of the vertically cut placenta from each embryo was used for Smart‐Seq3 library construction, and the other half for WGBS sequencing library construction. This way, all three layers of the placenta structure with different cell types were included in both sequencing methods. cDNA libraries for placenta and PGCs were generated using the Smart‐seq3 protocol. At E10.5 were 10–50 PGCs per dam sorted into lysis buffer, visual sex determination was performed at E13.5 and 5–50 PGCs/sex was sorted into lysis buffer for each dam for Smart‐seq 3 (Table S8, Supporting Information). Genotyping was performed to determine the sex of embryos from E10.5 and E13.5, and RNA from placenta of both E10.5 and E13.5 were extracted using Trizol method. The placenta RNA was pooled for sequencing according to sex and dam, except for DHT group at E10.5 stage where only 1 dam was pregnant, and the placenta was sequenced individually. RNA was reverse transcribed using Maxima H minus reverse transcriptase (Thermo Fisher). Resulting cDNA was amplified with KAPA HiFi HotStart ReadyMix (KAPA Biosystems) by 18 cycles of PCR, then libraries purified with 22% PEG Clean‐up beads. Quality check was performed using an Agilent 2100 BioAnalyser (Agilent Technology) to assess quality and quantity of the cDNA library. 100pg cDNA each sample was tagmented using a Tn5 transposase and amplified for 8 cycles using Nextera Index Primers (Ilumina Novaseq 6000).

### Smart‐Seq3 Bulk RNA Sequencing Mapping and Quantification

Raw demultiplexed fastq files were merged and processed using zUMIs^[^ ^76^ ^]^ (v2.9.7c) with STAR^[^ ^77^ ^]^ (2.7.10a) to generate expression profiles. Reads were mapped against GRCm38 and were quantified with gene annotations from GENCODE GRCm38.p6.

### Prime‐Seq Bulk RNA Sequencing Library Preparation

cDNA libraries for E13.5 liver were generated using the Prime‐Seq protocol.^[^ ^78^ ^]^ E13.5 liver RNA was isolated using a combination of TRIReagent (Sigma) and ReliaPrep RNA Miniprep Systems (Promega). Then liver RNA was pooled for sequencing according to sex and dam. The extracted RNA was normalized to a concentration of 10 ng uL^−1^, and 40 ng in total was used from each sample to provide the library. Reverse transcription was performed using Maxima H Minus Reverse Transcriptase (ThermoFisher Scientific), followed by preamplification using KAPA HiFi 2X ReadyMix (Roche). Thereafter, the obtained cDNA was normalized to a concentration of 6 ng uL^−1^. Subsequent steps were performed using the NEBNext Ultra II FS DNA Library Prep Kit (New England Biosciences), as described in the protocol for prime‐seq. Agilent Bioanalyzer 2100 High Sensitivity DNA Analysis Kits (Agilent) were used to QC both the cDNA and final library quality and fragment size.

### Prime‐Seq Bulk RNA Sequencing Mapping and Quantification

The adapters and polyG from Raw non‐demultiplexed fastq files were trimmed using cutadapt (v4.1) and fastp (0.23.2). The clean reads were processed using zUMIs^[^ ^76^ ^]^ (v2.9.7c) with STAR^[^ ^77^ ^]^ (2.7.10a) to generate expression profiles. To extract and identify the UMI‐containing reads in zUMIs, the base_definition: BC(1–12) and UMI (13–28) were specified for file 1 and cDNA (15–150) for file 2 in the YAML file. UMIs were collapsed using a Hamming distance of 1. Reads were mapped against GRCm38 and were quantified with gene annotations from Gencode GRCm38.p6.

### RNA Sequencing Analysis

From the RNA quantification matrices, we filtered out the low‐expressed genes. Genes were removed if they were expressed in the number of samples less than the average group size, i.e., total number of samples divided by group numbers. Principal component analysis and Spearman correlation were calculated based on log2 normalized variance stabilized transformed counts.

For differentially expressed analysis, the RNA quantification matrices were analyzed using DESeq2^[^ ^79^ ^]^ with the treatment condition as the variable of interest. Statistically significant genes (log2 foldchange >1, FDR < 0.05) were identified and then used for making plots and for downstream analysis. In addition, in the E10.5 placenta transcriptomic analysis, no difference was observed between male and female placenta (Table S9, Supporting Information), therefore analysis was performed without differentiating fetal sex.

For Cell type deconvolution analysis, the single nuclei,^[^ ^54^ ^]^ or single cell^[^ ^40^ ^]^ RNA count matrix from E10.5 placenta with the annotated cell type information and our RNA‐seq count matrix was processed using the CIBERSORTx^[^ ^80^ ^]^ to impute the placental or trophoblast cell type fractions.

For the genes that were identified in human genome‐wide association study and transcriptome‐wide association study, we leveraged the mouse human homologue information from Mouse Genome Informatics (http://www.informatics.jax.org/downloads/reports/HOM_MouseHumanSequence.rpt) and map the genes from human to mouse.

### Whole Genome Bisulfite Sequencing Library Preparation

At E10.5 50–200 PGCs per dam were sorted into lysis buffer, sex determination was performed at E13.5, and 25–500 PGCs/sex were sorted into lysis buffer for each dam for WGBS (Table S3, Supporting Information). Genotyping was performed to determine the sex of embryos from E10.5, and DNA from placenta of both E10.5 and E13.5 was extracted using QIAamp Fast DNA kit (Qiagen). Then placenta DNAs were pooled for sequencing according to sex and dam, except for DHT group at E10.5 stage where only 1 dam was pregnant, and the placenta were sequenced individually. 1 µg of genomic DNA each sample was spiked with 6.54 ng unmethylated lambda DNA then sonicated into a mean size of 350 bp fragments with ME220 Focused‐Ultrasonicator (Covaris). The ends of sonicated DNA fragments were repaired before adaptor ligation with NEBNext Ultra II DNA Library Prep kit for Ilumina (New England BioLabs) according to manufacturer's instruction. The adaptor ligated DNA was cleaned up with 0.8x AMPure xp (Beckman Coulter) beads to remove any adaptor dimer or small fragments. The ligated DNA was bisulfite converted with the EZ DNA Methylation‐Gold kit (Zymo Research) then PCR amplified for 10 cycles with KAPA HiFi Hotstart Uracil+ kit (Roche). 2% agarose gel was used to perform size selection of the amplified products and QIAquick Gel Extraction kit (Qiagen) to extract product of desired size.

### Whole Genome Bisulfite Sequencing Data Processing

For WGBS data, the illumina and library specific adapters were trimmed using bbduk (v38.98, BBMap – Bushnell B. – sourceforge.net/projects/bbmap/) with parameters ‘ktrim = r k = 23 mink = 11 hdist = 1 tpe tbo qtrim = rl trimq = 10 minlen = 2′. The reads after trimming were mapped to mouse genome GRCm38, deduplicated and methylated Cs were called using bismark. Coverage was calculated as ‘read length x number of uniquely mapped reads/2 652 783 500′ using a customed script. Differentially methylated sites and regions were analyzed using MethylSig package in R.^[^ ^81^ ^]^ Further annotation was made using AnnotationHub package In R.

### Statistical Analysis

To calculate the number of mice needed, power analysis was conducted based on our previous publication.^[^ ^6^, ^82^ ^]^ Based on 40.6% (SD 0.1) difference in AGD between androgen exposed and control mice, nine mice per group were needed to detect a significant difference (*P* < 0.05) with a power of 0.8. For all data obtained, Rout outlier test and Shapiro‐Wilk normality test are performed with Graphpad Prism (version 8.4.3) to check for outliers and data distribution before performing statistical analysis. Only exception was the serum hormone measurements for F0 dams, as LC‐MS is a very sensitive method and the readings obtained reflect the true value, so no outlier was removed for this dataset. For all F_0_ group comparisons were one‐way ANOVA with Dunnett post hoc test or Kruskal‐wallis test with Dunn's test used depending on if the data passes the Shapiro‐Wilk normality test. The DHT group was used to compare with CNT or DHT + FLU groups. All F_0_ statistics were done using GraphPad Prism (version 8.4.3) for Windows. For all embryo and F_1_ group comparisons, analysis of covariance (ANCOVA) was used whenever possible to adjust for litter as dam is considered as a covariate. ANCOVA statistics was performed in R (version 4.2.1) with “lme4” and “car” packages. Data are presented as mean ± s.e.m. Differences were considered statistically significant when *P* < 0.05.

## Conflict of Interest

The authors declare no conflict of interest.

## Author Contributions

H.L., H.J., and C.L. share first authorship and contributed equally to this work. H.L. designed the study, performed the mouse data collection, performed molecular analyses, analyzed the data, prepared the figures, prepared the sequencing library, and wrote the manuscript. H.J. analyzed the RNA and whole genome bisulfite sequencing data, prepared the figures, and was involved in manuscript preparation. C.L. was involved in the study design, preparation of the sequencing library, interpretation of the results, and preparation of the manuscript. E.D. was involved in the human organoid planning and revising of the manuscript. A.Z was responsible for prime seq library preparation. G.E. was involved in mouse experiment. HP.P. was involved in the embryo collection. S.R. performed molecular analysis. Y.P. was involved in the RNA sequencing library preparation. T.M. was involved in preparing human trophoblast stem cells. C.O. performed the serum sex steroid analyses with GC‐MS. E.L. performed the mouse embryo image data analyses. S.H. was involved in preparing human trophoblast stem cells and revising the manuscript. A.B. was involved in the study design, interpretation of the results, and preparation of the manuscript. E.S‐V. and Q.D. designed the study, analyzed the data, prepared the figures, and wrote the manuscript. All authors read and approved the final version of the manuscript.

## Supporting information

Supporting Information

Supporting Information

Supporting Information

Supporting Information

## Data Availability

The code and data used to produce the plots within this work will be released on the repository Dryad, https://doi.org/10.5061/dryad.47d7wm3mm upon publication of this manuscript. For peer review, the data can be downloaded via https://datadryad.org/stash/share/PUfveXmLV3WglNhdhH0_c2q_uetOAvRYHunEpFPhjsY.
