# Supplementary material for: Dissecting the Impact of Maternal Androgen Exposure on Developmental Programming through Targeting the Androgen Receptor
Source: Adv Sci (Weinh). 2024 Jul 29;11(36):2309429. doi: 10.1002/advs.202309429 (PMC11423211; doi:10.1002/advs.202309429)
Supplement: Supplementary file 1 — Supporting Information [file ADVS-11-2309429-s004.pdf]

ADVANCED SCIENCE
Open Access

Supporting Information

for *Adv. Sci.*, DOI 10.1002/advs.202309429

Dissecting the Impact of Maternal Androgen Exposure on Developmental Programming through Targeting the Androgen Receptor

Haojiang Lu, Hong Jiang, Congru Li, Emilie Derisoud, Allan Zhao, Gustaw Eriksson, Eva Lindgren, Han-Pin Pui, Sanjiv Risal, Yu Pei, Theresa Maxian, Claes Ohlsson, Anna Benrick, Sandra Haider, Elisabet Stener-Victorin and Qiaolin Deng**

Supporting Information

Dissecting the Impact of Maternal Androgen Exposure on Developmental Programming through Targeting the Androgen Receptor

Haojiang Lu[1,6], Hong Jiang[1,6], Congru Li[1,6], Emilie Derisoud[1], Allan Zhao[1], Gustaw Eriksson[1], Eva Lindgren[1], Han-Pin Pui[1,] Sanjiv Risal[1], Yu Pei[1], Theresa Maxian[2], Claes Ohlsson[3], Anna Benrick[4,5], Sandra Haider[2], Elisabet Stener-Victorin[1], Qiaolin Deng[1] **

*** Corresponding authors**

Elisabet Stener-Victorin
Karolinska Institutet, Biomedicum B5, Department of Physiology and Pharmacology, 171 77 Stockholm, Sweden, E-mail: elisabet.stener-victorin@ki.se
Qiaolin Deng
Karolinska Institutet, Biomedicum B5, Department of Physiology and Pharmacology, 171 77 Stockholm, Sweden, E-mail: qiaolin.deng@ki.se

This PDF file includes:

Fig. S1 Pregnancy phenotypes of peripubertal F0 dams and placenta.
Fig. S2 Flutamide prevent the major RNA expression and DNA methylation changes in placentae and offspring livers induced by excess androgen exposure.

Table S1 Sex distribution for survived embryo at E10.5 and E13.5.
Table S2 Information for samples used for RNA sequencing.
Table S3 Information for samples used for WGBS.
Table S4 E10.5 placenta DHT vs. CNT DEGs (1218 DEGs in total)_in separate file.
Table S5 Developmental phenotypes predicted by DHT vs. CNT DEGs_in separate file.
Table S6 Phenotype gene intersection.
Table S7 Decovolution results for E10.5 and E13.5 placenta RNA sequencing for identifying cell types.
Table S8 Number of PGCs collected during embryo dissection for RNA and WGBS sequencing. Table S9 Differentially expressed genes regulated by sex at E10.5_in separate file.

Figure S1

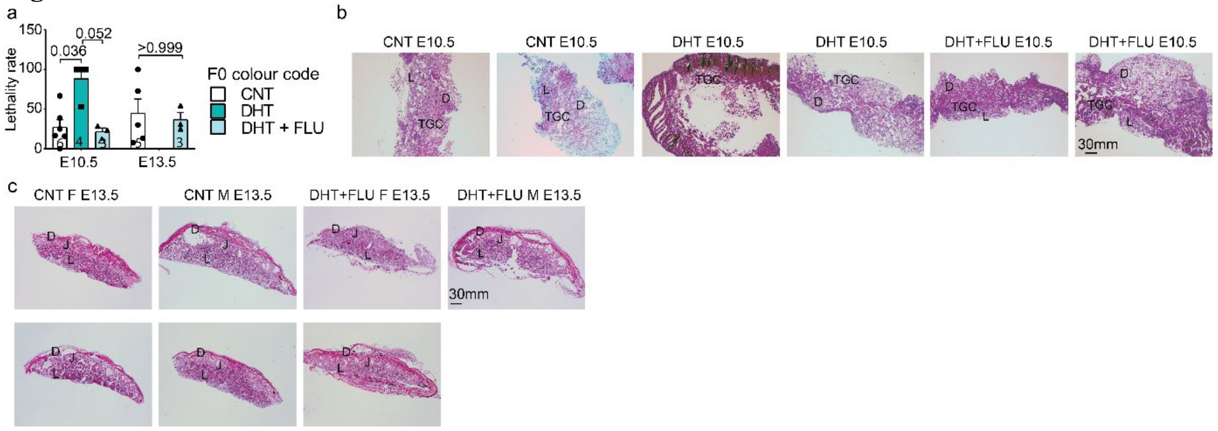

Fig. S1 Pregnancy phenotypes of peripubertal F0 dams and placenta

a, Total number of dead/absorbed embryos/dam at the time of dissection. Statistics performed by ANCOVA to control for litter. **b,** Representative bright-field images of H&E staining of E10.5 placentas. **c,** Representative bright-field images of H&E staining of E13.5 placentas TGC: trophoblast giant cell, L: labyrinth zone, J: junctional zone, D: decidua.

Figure S2

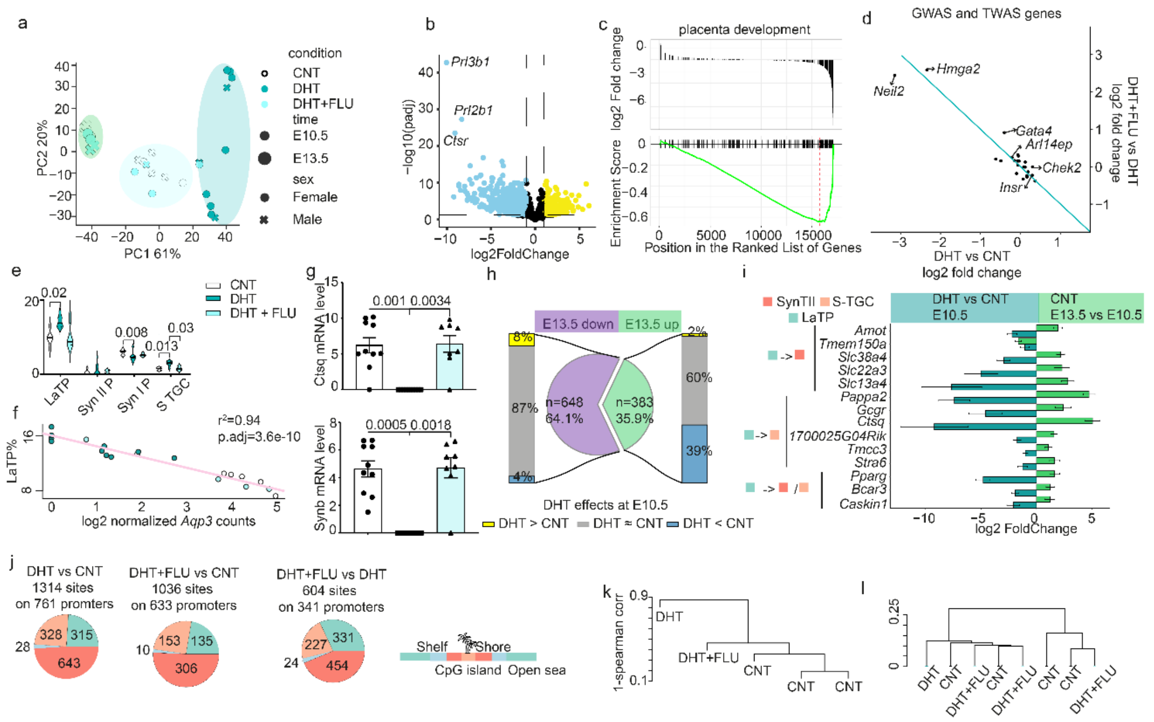

Fig. S2. Flutamide prevent the major RNA expression and DNA methylation changes in placentae and offspring livers induced by excess androgen exposure.
a, The first two principal components of gene expression matrices from the placenta samples. **b,** Vocalno plot of the gene expression from placentas at E10.5 comparing DHT with Control group. **c,** Gene set enrichment analysis reveals negative enrichment in placenta development in the placenta of DHT-implanted PCOS-mice compared to control mice. **d,** Scatter plots of fold changes of PCOS risk genes estimated with DESeq2. **e,** Cell type proportion deconvoluted aided by the single cell expression atlas for trophoblast at E10.5. **f,** Linear regression of *Aqp3* with labyrinth trophoblast precursors. P values are adjusted using the Holm method. **g,** Log normalized transcript counts of *Ctsq* and *Synb* in E10.5 placenta. **h,** The overlap between DEGs comparing DHT treated PCOS-mice and control mice at E10.5 or at E13.5. **i,** DEGs varied along the differentiation trajectory. The bar plot shows the fold change of genes at E10.5 or E13.5 by DHT treatment. The genes were the DEGs comparing DHT treated PCOS-mice and control mice at E10.5 or DEGs comparing E13.5 to E10.5. The genes were annotated with the differentiation trajectory if they were varied in the respective pseudo time trajectory is from. LaTP: labyrinth trophoblast progenitor, S-TGC: sinusoid trophoblast giant cells. **j,** Pie plots representing differentially methylated promoters' distribution on CpG annotations. **k,** Clustering with the distance metrics 1- spearman correlation from the gene expression in E10.5 PGC. **l,** Clustering of E10.5 PGC samples based on CpG methylation. In **e**, **f** and **j** data are represented as mean ± s.d. Statistics of **e** and **g** were performed with ANCOVA to control for litter. Number of dams used for RNA sequencing analysis: For embryonic stage E10.5, CNT=3, DHT=1, DHT+FLU=3. For embryonic stage E13.5, CNT =3, DHT+FLU=3.

Table S1 **Sex distribution for survived embryo at E10.5 and E13.5**.

E10.5	Female	Male
CNT 1	8	6
CNT 2	4	7
CNT 3	1	0
CNT 4	9	5
CNT 5	1	2
CNT 6	10	4
	57.90%	42.10%
DHT	8	4
	66.70%	33.30%
FLU 1	6	4
FLU 2	9	2
FLU 3	1	3
	64%	36%

E13.5	Female	Male
CNT 1	6	6
CNT 2	7	6
CNT 3	4	9
	44.70%	55.30%
FLU 1	6	3
FLU 2	6	4
FLU 3	5	4
	60.70%	39.30%

Table S2 **Information for samples used for RNA sequencing**.

Tissue	Stage	Treatment	Gender	Replicates	Average detected genes
Placenta	E10.5	CNT	Female	2	17660
			Male	4	18133
		DHT	Female	7	16844
			Male	3	16310
		DHT+FLU	Female	3	18479
			Male	1	20158
	E13.5	CNT	Female	2	16595
			Male	2	16721
		DHT+FLU	Female	2	17797
			Male	2	16908
PGC	E10.5	DHT+FLU	\	2	13620
	E13.5	CNT	Female	4	14935
			Male	2	12325
		DHT+FLU	Female	2	12212
			Male	1	15315

Table S3 **Information for samples used for WGBS**.

Tissue	Stage	Treatment	Gender	Replicates	**Mean cov***	**% mCpG**	**M C's**	**% Dups**	**% Aligned**	**M Unique**
Placenta	E10.5	CNT	Female	4	4.28	61.20%	1571.63	52.80%	73.83%	31.65
			Male	4	4.11	60.33%	1865.32	51.44%	75.27%	37.51
		DHT	Female	9	4.08	64.20%	841.54	67.05%	48.75%	16.14
			Male	3	3.19	62.60%	1128.44	64.54%	58.12%	22.02
		DHT+FLU	Female	3	6	56.70%	1518.04	51.98%	67.04%	29.88
			Male	3	6.2	55.80%	1318.22	55.08%	66.98%	26.19
	E13.5	CNT	Female	2	3.44	48.70%	629.54	65.07%	36.28%	14.05
			Male	3	3.67	47.74%	747.89	61.13%	43.64%	11.16
		DHT+FLU	Female	1	2.23	52.20%	513.72	54.88%	23.56%	8.77
			Male	1	3.95	46.70%	904.72	54.32%	43.36%	16.54
PGC	E10.5	CNT	\	4	1.65	68.90%	924.975	48.88%	56.83%	15.93
		DHT	\	1	1.2	72.60%	656.3	27.30%	55.90%	11.3
		DHT+FLU	\	3	1.4	68.27%	784.2	37.40%	54.53%	13.53
	E13.5	CNT	Female	2	1.85	13.35%	984	35.40%	56.30%	17.6
			Male	2	2.1	16.25%	1155.9	33.75%	57.45%	19.7
		DHT+FLU	Female	3	1.73	18.27%	988.0333333	42.93%	58.63%	16.4
			Male	2	1.7	15.30%	958.3	55.20%	63.20%	15.75

Table S6 Phenotype gene intersection

mgi_symbol	description	Abnormal pla	Abnormal br	Anxiety o	Insulin level, release, sensitivity, resist
**Pparg**	peroxisome proliferator activated receptor gamma [Source:MGI Symbol;Acc:MGI:97747]	1	1	0	1
**Cdh1**	cadherin 1 [Source:MGI Symbol;Acc:MGI:88354]	1	0	0	1
**Gab1**	growth factor receptor bound protein 2-associated protein 1 [Source:MGI Symbol;Acc:MGI:108088]	1	0	0	1
**Hmox1**	heme oxygenase 1 [Source:MGI Symbol;Acc:MGI:96163]	1	0	0	1
**Nos1**	nitric oxide synthase 1, neuronal [Source:MGI Symbol;Acc:MGI:97360]	0	1	0	1
**Nos2**	nitric oxide synthase 2, inducible [Source:MGI Symbol;Acc:MGI:97361]	0	1	0	1
**Stra6**	stimulated by retinoic acid gene 6 [Source:MGI Symbol;Acc:MGI:107742]	0	1	0	1
**Tcf7l2**	transcription factor 7 like 2, T cell specific, HMG box [Source:MGI Symbol;Acc:MGI:1202879]	0	1	0	1
**Apoa2**	apolipoprotein A-II [Source:MGI Symbol;Acc:MGI:88050]	0	0	0	1
**Arnt2**	aryl hydrocarbon receptor nuclear translocator 2 [Source:MGI Symbol;Acc:MGI:107188]	0	0	0	1
**Bcar3**	breast cancer anti-estrogen resistance 3 [Source:MGI Symbol;Acc:MGI:1352501]	0	0	0	1
**Ceacam2**	CEA cell adhesion molecule 2 [Source:MGI Symbol;Acc:MGI:1347246]	0	0	0	1
**Cxcr1**	chemokine (C-X-C motif) receptor 1 [Source:MGI Symbol;Acc:MGI:2448715]	0	0	0	1
**Fabp3**	fatty acid binding protein 3, muscle and heart [Source:MGI Symbol;Acc:MGI:95476]	0	0	0	1
**Fabp5**	fatty acid binding protein 5, epidermal [Source:MGI Symbol;Acc:MGI:101790]	0	0	0	1
**Fstl3**	follistatin-like 3 [Source:MGI Symbol;Acc:MGI:1890391]	0	0	0	1
**Gcgr**	glucagon receptor [Source:MGI Symbol;Acc:MGI:99572]	0	0	0	1
**Ghrh**	growth hormone releasing hormone [Source:MGI Symbol;Acc:MGI:95709]	0	0	0	1
**Gucy2c**	guanylate cyclase 2c [Source:MGI Symbol;Acc:MGI:106903]	0	0	0	1
**Hmga1**	high mobility group AT-hook 1 [Source:MGI Symbol;Acc:MGI:96160]	0	0	0	1
**Irx3**	Iroquois related homeobox 3 [Source:MGI Symbol;Acc:MGI:1197522]	0	0	0	1
**Lin28a**	lin-28 homolog A [Source:MGI Symbol;Acc:MGI:1890546]	0	0	0	1
**Noct**	nocturnin [Source:MGI Symbol;Acc:MGI:109382]	0	0	0	1
**Pcbd1**	pterin 4 alpha carbinolamine dehydratase/dimerization cofactor of hepatocyte nuclear factor 1 alpha (TCF1) 1 [S	0	0	0	1
**Pfkm**	phosphofructokinase, muscle [Source:MGI Symbol;Acc:MGI:97548]	0	0	0	1
**Pik3cb**	phosphatidylinositol-4,5-bisphosphate 3-kinase catalytic subunit beta [Source:MGI Symbol;Acc:MGI:1922019]	0	0	0	1
**Ppp1r3g**	protein phosphatase 1, regulatory subunit 3G [Source:MGI Symbol;Acc:MGI:1923737]	0	0	0	1
**Psg27**	pregnancy-specific beta-1-glycoprotein 27 [Source:MGI Symbol;Acc:MGI:1891359]	0	0	0	1
**Rarres2**	retinoic acid receptor responder (tazarotene induced) 2 [Source:MGI Symbol;Acc:MGI:1918910]	0	0	0	1
**Sgms2**	sphingomyelin synthase 2 [Source:MGI Symbol;Acc:MGI:1921692]	0	0	0	1
**Sgpp2**	sphingosine-1-phosphate phosphatase 2 [Source:MGI Symbol;Acc:MGI:3589109]	0	0	0	1
**Slc7a5**	solute carrier family 7 (cationic amino acid transporter, y+ system), member 5 [Source:MGI Symbol;Acc:MGI:12	0	0	0	1
**Sox9**	SRY (sex determining region Y)-box 9 [Source:MGI Symbol;Acc:MGI:98371]	0	0	0	1
**Tg(Ins1-Tag**	NA	0	0	0	1

Overlapping test

	Abnormal Placenta development (n=67)		
	intersection size	Overlapping p-value	Jaccard Index
Abnormal brain development (n=71)	14	0.0014	0.1
Anxiety or Depression (n=18)	0	1	0
Insulin level, release, sensitivity, resist (n=34)	4	0.33	0

Table S7 Decovolution results for E10.5 and E13.5 placenta RNA sequencing for identifying cell types

Mixture	LaTP 2	Glycogen Cells	LaTP	JZP 1	SynTI	SynTI Precursor	JZP 2	SpT Precursor	SynTII	SpT	SynTII Precursor	S-TGC	S-TGC Precursor	Decidual Stroma	Endothelial	Blood	P-value	Correlation	RMSE	Absolute score (sig.score)
CNTF1_10.5	0	0	0	0	0	0	13.3191400493258	19.4461194803244	6.31657960856648	1.22692436572291	0	0	51.5263832208949	20.7037272698037	0	0	0.11	0.0377214773664031	1.08597476046329	112.538873994638
CNTM1_10.5	0	0	0	0	0	0	10.1767807837971	14.915798857379	6.56113820765902	2.38543914175618	0	0	34.1943499443918	10.494566600061	0	0	0.11	0.0383339817811306	1.06732447456173	78.728073535044
CNTM2_10.5	0	0	0	0	0	0	2.81152304021764	10.1233756687611	2.77863584560861	0	0	0.100264989622464	13.8235350638988	14.7992797159052	0	0	0.044	0.057363605509218	1.17475876020846	44.4366143240138
CNTF2_10.5	0	0	0	0	0	0	3.65547755303774	5.32196720511833	2.1421595323828	0.0135113119327887	0	0	9.8656526875638	8.25630639399365	0	0	0.086	0.0434911823920923	1.1399766686159	29.2550746840291
CNTM3_10.5	0	0	0	0	0	0	10.4047571585776	14.3951552496601	5.41897650700816	4.59683365039327	0	0	32.1598492712007	23.9983845017277	0	0	0.094	0.0404648818077466	1.13607456755063	90.9739563385676
CNTF3_10.5	0	0	0	0	0	0	4.54329821076606	10.054294212692	7.70068896686145	3.62748409272246	0	0	19.5543640649103	33.6853243011477	0	0	0.086	0.043245549468682	1.27222860507768	79.1654538491
CNTM4_10.5	0	0	0	0	0.121688794814597	0	7.62501537977332	16.6692511039995	7.80094823014916	0.0516977745485044	0	0	24.2593933368026	27.0847974136158	0	0	0.036	0.0643044391399872	1.1613629842428	83.6127920337036
DHTF1_10.5	0	0	0	0	0	0	0	3.6839801000812	2.28092032938966	0.822765296315362	0	0	9.64471133448249	21.3837853296202	0	0	0.246	0.0219380882156952	1.4446402855801	37.8161623898889
DHTF2_10.5	0	0	0	0	0	0	8.60656032572333	0	7.72849487078014	8.9239344159632	0	0	14.6051883712238	58.4127274165393	0	0	0.026	0.0719801107475712	1.44778563063143	98.2769054002298
DHTM1_10.5	0	0	0	0	0	0	0.117007640736129	2.37316877052075	10.4011177908201	5.0390414756749	0	0	14.1359349166673	35.431622932965	0	0	0.152	0.0322852310645127	1.39388103339945	67.4978935273842
DHTF3_10.5	0	0	1.82751757480754	0	0	0	0.395166353835177	3.76838568549616	2.19914435871633	0	0	0	5.74387629808109	36.7342360408217	0	0	0.082	0.0446229795289822	1.63683795530698	50.6683263117579
DHTM2_10.5	0	0	0.430659803585032	0	0	0	3.41677581208561	2.90833968638282	2.81502880996672	0	0	0	6.79298780470579	30.6308461529791	0	0	0.054	0.0544636524285719	1.52792296185913	46.9946380697051
DHTF4_10.5	0	0	6.6861801122993	0	0	0	0	9.92097910319257	10.2443094948607	0	0	0	10.1050953733796	34.8588323161146	0	0	0.162	0.0313558248023796	1.33941820148318	71.8153963998468
DHTM3_10.5	0	0	0	0	0	0	0	15.3806894243365	1.16469678874899	0	0	0	12.8303408136566	17.4132427166512	0	0	0.17	0.0301204044352305	1.23296026151908	46.7889697433933
DHTF5_10.5	0	0	0	0	0	0	0.905605923138651	1.26679823424262	10.1444207146048	2.77270111814063	0	0	20.2732054248366	33.0577971181658	0	0	0.092	0.0413473712147712	1.33361520985202	68.4205285331291
DHTF6_10.5	0	0	0	0	0	0	6.32815605821805	0	4.53683071324058	5.17788909019678	0	0	14.9408295602492	32.7053026209909	0	0	0.122	0.0355837622978366	1.37424771462463	63.6890080428954
DHTF7_10.5	0	0	10.6439904437735	0	0	0	0	19.7063993635226	0.980249304658643	0	0	0	15.971485205235	26.2834750700181	0	0	0.342	0.0145711103875874	1.21887577723048	73.585599387208
DHTF8_10.5	0	0	0	0	0	0	0	1.94857812345807	4.61338475827872	3.54890982256158	0.050432311095031	0	12.8622640131665	28.7113984168634	0	0	0.032	0.0684925843819979	1.40115156493834	51.7349674454232
FLUF1_10.5	0	0	0	0	0	0	20.2092920009942	11.3095880835407	7.24136014406598	2.28401269213772	0	0	38.2752179921004	14.5415018945145	0	0	0.226	0.0238502983546371	1.07605988647818	93.8609728073535
FLUM1_10.5	0	0	0	0	0	0	20.0343081871849	24.5164509024037	8.3918625522463	3.98604485199611	0	0	55.8889838956441	19.7832687985755	0	0	0.166	0.0305745851017175	1.07608811099357	132.600919188051
FLUF2_10.5	0	0	0	0	0	0	8.64044722344073	11.2473204381811	5.04367957433996	4.28589553993457	0	0	41.8755356367137	11.3176922499713	0	0	0.27	0.0197431161489705	1.08004240011083	82.4105706625814
FLUM2_10.5	0	0	0.000770019085189426	0	0	0.00015484567852251	0.000634480312770676	0	7.95485389636729e-05	7.51711738743136e-05	9.8217764362573e-05	0	0.000485687572705181	0	0	0	0.338	0.0151035034195718	1.03754742344578	0.00229797012638836
FLUF3_10.5	0	0	0	0	0	0	10.5261757802567	7.97781733472686	9.44151548761892	1.02604857882107	0	0	17.2744562197398	49.5506454729964	0.0218014858555811	0	0.028	0.0718280958038007	1.34678065808501	95.8184603600153
CNTF1_13.5	0	3.94102398252613	0	0	4.49033128885875	0	0	1.80234309976267	11.085071286737	46.1378437577124	0	0	18.630783076796	4.81953571748837	0	0	0.002	0.136160369780862	1.26081812810136	90.9069322098813
CNTM1_13.5	0	2.42960921490895	0	0	1.24876602252962	0	0	0	3.69628595861475	21.2781382168075	0	0	6.37453535562277	0.722952477782264	0	0	0.002	0.134940153121316	1.34715841666651	35.7502872462658
CNTF2_13.5	0	1.20025368949835	0	0	0.345394487324901	0.156521240167054	0	0	1.30280984176361	10.5573843934257	0.197356787878912	0	1.70654587776898	0.251849346668842	0	0	0.004	0.124963776281053	1.44222796775243	15.7181156644964
CNTF3_13.5	0	8.09539769715682	0	0	7.61735749582572	0	0	0	12.7235258608909	49.9584987924096	0	6.83956791497198	10.6546014712535	2.21407642815794	0	0	0.002	0.142192102993437	1.25784669514528	98.1030256606664
CNTM2_13.5	0	10.4071184960548	0	0	2.97660446710173	0	0	0	10.3351890734682	40.0549921650411	0	4.80538702612245	10.2334041279226	1.9023563486169	0	0	0.002	0.159720175730705	1.2399904756452	80.7150517043278
FLUF1_13.5	0	1.82467918683293	0	0	1.12855959051096	0	0	1.52287424289318	4.20511158583052	25.8217948344579	0	0.549887283589704	5.95260828954573	1.68425901927664	0	0	0.002	0.157769201831686	1.34711271294444	42.6897740329376
FLUM1_13.5	0	1.34416900614519	0	0	0	0	0	0	0.51139160679107	10.4503628408769	0.116347413120752	0	1.60340129742019	0.188805047442167	0	0	0.002	0.160137131515655	1.4935289068251	14.2144772117962
FLUF2_13.5	0.0428447061775359	0.0284391265689087	0	0	0.0161726734901412	0	0	0	0.0292164933937236	0.858942916408446	0.039761943319377	0	0.0910947564978618	0	0	0	0.004	0.126870639685101	1.55498627984464	1.10647261585599
FLUF1_13.5.1	0	1.40726593825083	0	0	6.98014860074895	0	0	3.33176961908228	11.9962818804395	55.1429526134321	0	5.2520176289734	20.7858514285643	2.9413224015772	0	0	0.00800000000000001	0.104830843209116	1.28317127273728	107.837610111069
FLUM3_13.5	0	1.81113392580219	0	0	2.12086602924178	0	0	27.3952188783577	12.5181759068293	55.7358893629276	0	8.36796447015201	4.87177650904764	11.0054781730994	0	0	0.004	0.132988061339375	1.26497158586388	123.826503255458
FLUM2_10.5.1	0	13.8778424148529	0	0	0	0	0	32.5961443124338	9.43978950882203	2.75269034012667	0	0	23.4673913389091	23.460933352569	0	0	0.204	0.0257187083007453	1.13024298761784	105.594791267714
FLUM2_13.5	0	0	0	0	0.304029027165256	0	6.15061844810143	16.0008018301499	7.92029215739919	34.469250390962	0	0.554040631267618	11.8365082549925	8.4848728945848	0	0	0.004	0.130373252308627	1.2134898894469	85.7204136346228

Table S8 **Number of PGCs collected during embryo dissection for RNA and WGBS sequencings**

E10.5	Dam ID	No. PGC for WGBS	No. of PGC for RNA seq
	1	60	0
	2	200	100
	3	50	
	4	500	50
CNT	5	500	50
	1	200	32
	1	100	50
	2	200	34
FLU	3	200	50

E13.5	Dam ID	No. PGC for WGBS from Female embryo	No. PGC for WGBS from Male embryo	No. of PGC for RNA seq from Female embryo	No. of PGC for RNA seq from Male embryo
	1	500	400	50	50
	2	200	200	50	50
CNT	3	500	500	50	50
	1	110	166	16	22
	2	105	25	10	5
FLU	3	100	50	10	10
